# Peroxynitrite‐induced structural remodeling and aggregation of hemoglobin under nitroxidative stress

**DOI:** 10.1002/pro.70762

**Published:** 2026-08-12

**Authors:** Shaik Basha, Aradhika Vijeev, Darshan Chikkanayakanahalli Mukunda, Krishna Kishore Mahato

**Affiliations:** ^1^ Department of Biophysics Manipal School of Life Sciences, Manipal Academy of Higher Education Manipal India; ^2^ Department of Biosciences & Bioengineering Indian Institute of Technology Dharwad Dharwad Karnataka India

**Keywords:** amyloid, hemoglobin, nitroxidative stress, peroxynitrite, protein aggregation

## Abstract

Peroxynitrite (PN), generated by the reaction of nitric oxide with superoxide, is a potent reactive nitrogen species (RNS) implicated in nitroxidative protein damage in cardiovascular and neurodegenerative diseases. Hemoglobin (Hb), the principal oxygen‐transport protein of erythrocytes, is a key intravascular scavenger and target of PN; however, how controlled PN exposure translates into structural remodeling and aggregation of Hb remains poorly understood. Purified human Hb was exposed to PN across sub‐stoichiometric to moderate oxidant excess and characterized using intrinsic fluorescence, UV–visible (UV–vis) spectroscopy, 2,4‐dinitrophenylhydrazine (DNPH) carbonyl assay, Thioflavin T (ThT), 8‐anilino‐1‐naphthalenesulfonic acid (ANS), Congo Red (CR), Fourier‐transform infrared (FTIR) spectroscopy with Amide I deconvolution, dynamic light scattering (DLS) with zeta potential, x‐ray powder diffraction (XRPD), scanning electron microscopy (SEM), atomic force microscopy (AFM), and fluorescence microscopy. At low oxidant loads, PN caused concentration‐dependent fluorescence quenching, carbonyl accumulation, and graded UV–visible absorbance increases, consistent with heme‐centered oxidation and limited aromatic residue modification. At higher concentrations, Amide I deconvolution revealed collapse of *α*‐helical content from 66.41% to 8.81% and *β*‐sheet enrichment to 71.18%, accompanied by surface charge neutralization, increased hydrodynamic diameter, and enhanced nanoscale roughness. Despite *β*‐sheet accumulation and ThT/CR‐positive aggregate formation, XRPD, AFM, SEM, and fluorescence microscopy showed no long‐range crystalline order or fibrillar morphology, demonstrating that PN drives Hb toward amorphous and oligomeric assemblies rather than canonical amyloid fibrils. Time‐dependent assays at 37°C confirmed that PN modification lowers the nucleation barrier for thermally driven aggregation. These findings define physicochemical thresholds separating protective scavenging from structural destabilization and offer a framework for distinguishing nitroxidative protein aggregation from classical amyloid fibrillation.

## 1 INTRODUCTION

Reactive nitrogen species, particularly the anion–acid pair oxoperoxonitrate(1−)/hydrogen oxoperoxonitrate (commonly referred to as peroxynitrite), are major mediators of oxidative and nitrative protein modifications implicated in neurodegenerative (Basha et al., 2023; Basha, Mukunda, et al., 2025; Basha, Nadkarni, et al., 2026; Basha, Pranavi, et al., 2025), cardiovascular (Choraria et al., 2025), and inflammatory diseases (Agarwal et al., 2018; Arif et al., 2017; Smallwood et al., 2025). Peroxynitrite (PN) is generated *in vivo* by the diffusion‐controlled reaction of nitric oxide with superoxide (1–2 × 10^10^ M^−1^ s^−1^), making its formation kinetically favored under conditions of elevated nitric oxide flux. Its reactivity is strongly influenced by pH and carbon dioxide, which promote the formation of the ONOOCO_2_^−^ adduct and secondary radicals such as nitrogen dioxide and carbonate radicals (Kalinga, 2006; Pietraforte et al., 2003; Romero et al., 2003). These reactive intermediates oxidize thiols and transition‐metal centers and nitrate aromatic residues in proteins and nucleic acids, leading to structural and functional alterations that serve as markers of nitrosative stress (Agarwal et al., 2018; Kosmachevskaya, Nasybullina, Shumaev, Novikova, & Topunov, 2021; Romero et al., 2003).

Hemoglobin (Hb), the major oxygen‐transport protein of erythrocytes, is both a primary intravascular target and an efficient scavenger of PN (Agarwal et al., 2018; Alayash et al., 1998; Boccini & Herold, 2004; Kosmachevskaya, Nasybullina, Shumaev, Chumikina, et al., 2021; Minetti et al., 2000; Smallwood et al., 2025). Oxyhemoglobin reacts rapidly with PN (≈10^4^ M^−1^ s^−1^), promoting nitrate formation and oxidation of ferrous heme to methemoglobin with only limited generation of ferryl intermediates and protein‐centered radicals (Basha, Vijeev, et al., 2026; Herold & Fago, 2005; Kalinga, 2006; Romero et al., 2003). Methemoglobin and metmyoglobin can further catalyze PN isomerization through transient iron(III)‐PN complexes, whereas deoxyhemoglobin reacts even faster under hypoxic conditions (Herold & Fago, 2005; Kalinga, 2006).

Despite its high oxidizing potential, PN induces relatively low levels of tyrosine nitration in Hb, highlighting the predominant scavenging role of the heme center (Herold & Fago, 2005; Kalinga, 2006; Kosmachevskaya, Nasybullina, Shumaev, Novikova, & Topunov, 2021; Pietraforte et al., 2003). Tyrosine nitration remains limited even under oxidant excess and depends on the PN:Hb ratio, carbon dioxide, and accessibility of the heme pocket (Pietraforte et al., 2003). At higher oxidant concentrations, PN also promotes tyrosyl and cysteinyl radical formation, protein crosslinking, and altered peroxidase‐like activity, whereas lower doses produce only modest functional changes without extensive structural disruption (Pietraforte et al., 2003; Romero et al., 2003). However, how these molecular events translate into mesoscopic structural remodeling, early aggregation, *β*‐sheet enrichment, and the formation of ordered or non‐ordered aggregates remains poorly understood.

To address this gap, the present study investigates the structural integrity and aggregation behavior of hemoglobin (Hb) following controlled PN exposure. Purified Hb (15 μM) was treated with PN (5–100 μM; 0.3–6.7‐fold molar excess), representing sub‐stoichiometric to moderate oxidant excess relevant to acute oxidative stress. PN‐induced structural remodeling and aggregation were then evaluated using complementary spectroscopic, biophysical, and microscopic approaches to establish how PN influences Hb conformation and aggregate morphology (Arif et al., 2017; Minetti et al., 2000; Zuercher et al., 2025). By integrating these complementary approaches, this study investigates whether Hb functions predominantly as a protective PN scavenger or undergoes progressive conformational destabilization and aggregation under controlled low‐to‐moderate oxidant excess. Particular emphasis is placed on distinguishing amorphous and oligomeric aggregates from highly ordered amyloid fibrils. Defining this balance between protective scavenging and structural destabilization provides insight into Hb behavior during nitroxidative stress and establishes an experimental framework for future studies on redox‐regulated Hb function and the development of protective strategies, including endogenous and synthetic PN scavengers such as dinitrosyl iron complexes and glutathione derivatives (Agarwal et al., 2018; Alayash et al., 1998; Boccini & Herold, 2004; Kosmachevskaya, Nasybullina, Shumaev, Chumikina, et al., 2021; Minetti et al., 2000; Smallwood et al., 2025).

## 2 RESULTS

### 2.1 Microenvironment alterations of endogenous fluorophores

**FIGURE 1:**
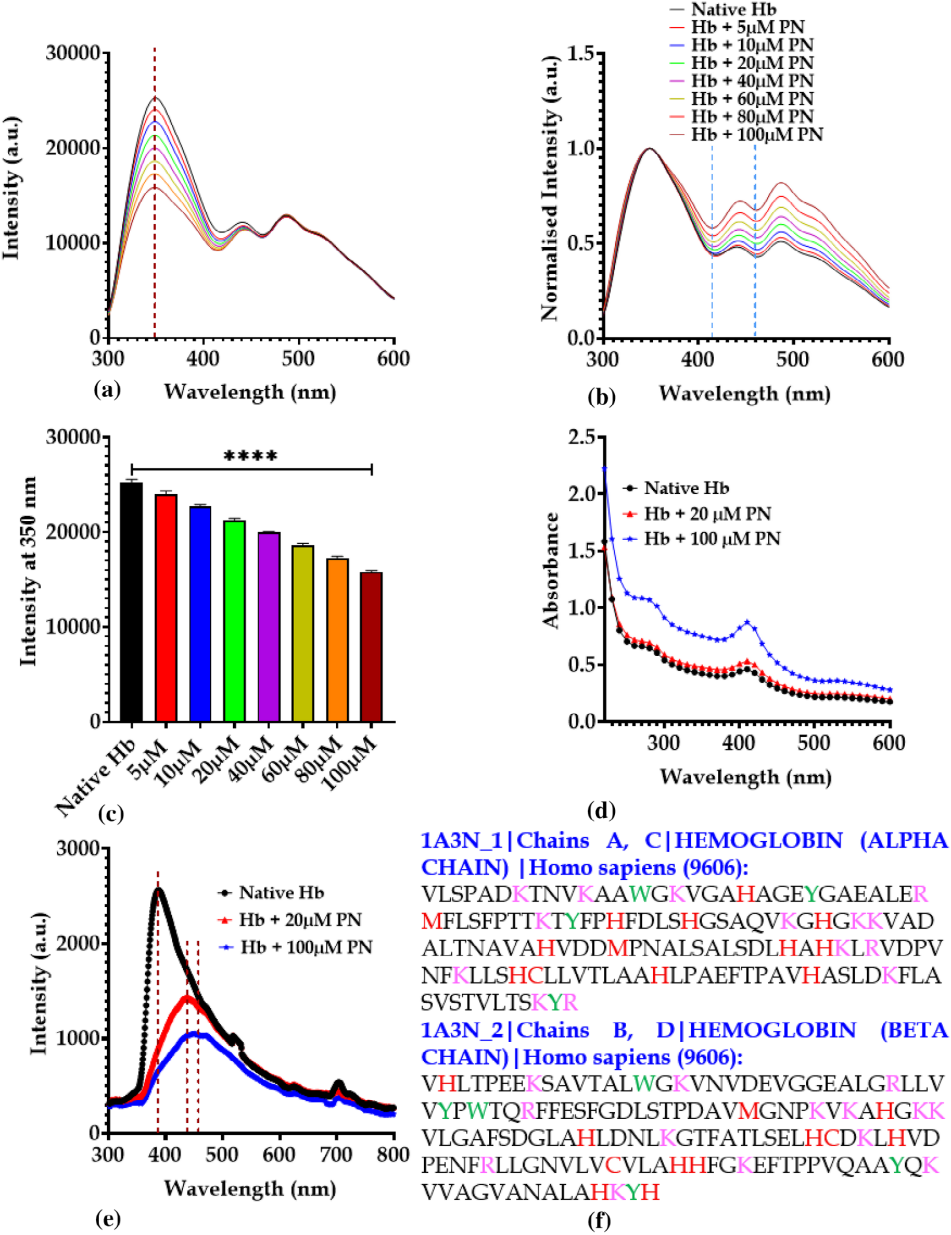
Peroxynitrite‐induced quenching of intrinsic hemoglobin fluorescence and PN‐susceptible residues in the globin chains. (a) Steady‐state fluorescence emission spectra of hemoglobin (15 μM) excited at 285 nm in the absence and presence of PN with increasing PN concentrations (5–100 μM); (b) The same spectra normalized to the native maximum illustrate that quenching is monotonic across the 300–600 nm region and does not introduce distinct new long‐wavelength bands; (c) Fluorescence intensity at 350 nm (mean ± SD, *n* = 3) decreases progressively with PN concentration; one‐way ANOVA with appropriate post‐hoc tests indicates that all PN‐treated groups differ significantly from native Hb (****p< 0.0001); (d) UV–visible absorbance scan of native Hb and PN‐treated Hb in the spectral region 220–600 nm; (e) The fluorescence spectra of native Hb and PN‐treated Hb were recorded in the spectral range of 300–800 nm upon excitation at 325 nm and (f) Amino‐acid sequences of human *α*‐ and *β*‐globin chains (PDB 1A3N), with PN‐susceptible residues highlighted: Aromatic Trp/Tyr (green), positively charged Lys/Arg (magenta), and redox‐active His/Met/Cys (red), indicating multiple potential sites for PN‐mediated oxidation and nitration within the hemoglobin tetramer. The sequence map is included only to illustrate possible interaction sites and does not represent experimentally verified Hb–PN binding residues or adduct assignments in the present study.

The intrinsic fluorescence measurements (Figure 1a–c) demonstrated that PN caused a progressive and statistically significant decrease in the fluorescence of Hb, consistent with PN‐induced perturbation of the microenvironment and quenching of aromatic residues under the conditions tested. Upon excitation at 285 nm, native Hb displayed a dominant emission band with a maximum near 340–350 nm (Figure 1a), characteristic of intrinsic tryptophan (Trp) fluorescence, with β37Trp representing the major contributor in adult HbA, along with minor contributions from other aromatic residues. Incremental addition of PN (5–100 μM) resulted in a monotonic decrease in the intensity of this band without an appreciable shift in the emission maximum, indicating that PN treatment primarily quenched intrinsic aromatic fluorescence without causing substantial changes in the average polarity of the Trp microenvironment detectable by steady‐state fluorescence. In addition to the main 340–350 nm emission band, the spectra exhibited a broad shoulder extending into the 400–480 nm region, with characteristic local maxima highlighted by the vertical dashed lines (Figure 1b). The normalized spectra (Figure 1b) further demonstrated that the overall spectral profile was largely preserved over the 300–600 nm emission range, and that quenching progressed smoothly with increasing PN concentration without introducing distinct new long‐wavelength bands. Although minor differences between individual traces were observed at wavelengths above 400 nm, no separate, well‐resolved additional emission bands were evident in this region. Consistent with the spectral traces, the bar plot of emission intensity at 350 nm (Figure 1c) demonstrated a concentration‐dependent reduction in fluorescence, and Welch's ANOVA indicated that all PN‐treated groups differ significantly from native Hb (****p< 0.0001), supporting a concentration‐dependent quenching response with increasing PN concentration.

### 2.2 UV–visible absorption spectroscopy

The UV–visible spectra (Figure 1d) revealed that PN treatment increased the absorbance of Hb across the 220–500 nm range in a concentration‐dependent manner, indicating PN‐induced modifications in the heme environment and oxidation state without substantial spectral rearrangements. In the far‐UV region (<300 nm), both 20 and 100 μM PN produced higher absorbance than native Hb, suggesting alterations in the aromatic side‐chain and peptide‐backbone environment that may reflect modest conformational or electronic changes linked to the heme group. In the near‐UV/visible region (~280–420 nm), PN‐treated samples showed a gradual rise in absorbance, with the 100 μM PN spectrum displaying the largest increase and a more noticeable shoulder near 400–420 nm, whereas the native spectrum remained comparatively featureless in this range. The difference between 20 μM and 100 μM was characterized primarily by a graded increase in absorbance and broadening of the existing bands, rather than the appearance of new, sharply defined peaks.

### 2.3 Oxidative modifications in hemoglobin

The oxidative modifications of PN‐treated Hb were further evaluated using fluorescence measurements upon excitation at 325 nm; the emission spectra of native and PN‐treated Hb showed clear PN‐dependent changes in both band position and intensity. Native Hb (Figure 1e) exhibited a strong emission peak near 390 nm. Upon PN treatment, this band decreased markedly in intensity and shifted toward longer wavelengths, with new emission maxima appearing around 442 nm and 462 nm in both 20 and 100 μM PN‐treated samples. A very weak, broad emission feature near 700 nm was also observed in PN‐treated samples. These spectral changes indicate PN‐induced structural and chemical modifications in Hb. The sequence map (Figure 1f) highlights the distribution of aromatic (Trp/Tyr), cationic (Lys/Arg), and redox‐sensitive (His/Met/Cys) residues within the *α*‐ and *β*‐globin chains, illustrating multiple potential sites for PN‐mediated oxidation and nitration. However, the present steady‐state fluorescence data do not permit the assignment of these spectral changes to specific Trp/Tyr residues or individual PN‐derived covalent modifications. Therefore, the precise PN–residue interactions cannot be conclusively established from the present data.

### 2.4 Determination of carbonyl content

The DNPH assay demonstrated a clear dose‐dependent increase in carbonyl formation in Hb upon PN treatment, as summarized in Table S1 (Supporting Information). Native (untreated) Hb exhibited carbonyl content of 0.00 ± 0.00 nmol/mg protein, indicating no detectable backbone carbonyls under the assay conditions. Following treatment with 20 μM PN, carbonyl levels rose to 0.66 ± 0.02 nmol/mg protein, and exposure to 100 μM PN further increased carbonyl content to 1.79 ± 0.03 nmol/mg protein. These results demonstrated a concentration‐dependent increase in protein carbonyl formation following PN treatment.

### 2.5 Thioflavin T

**FIGURE 2:**
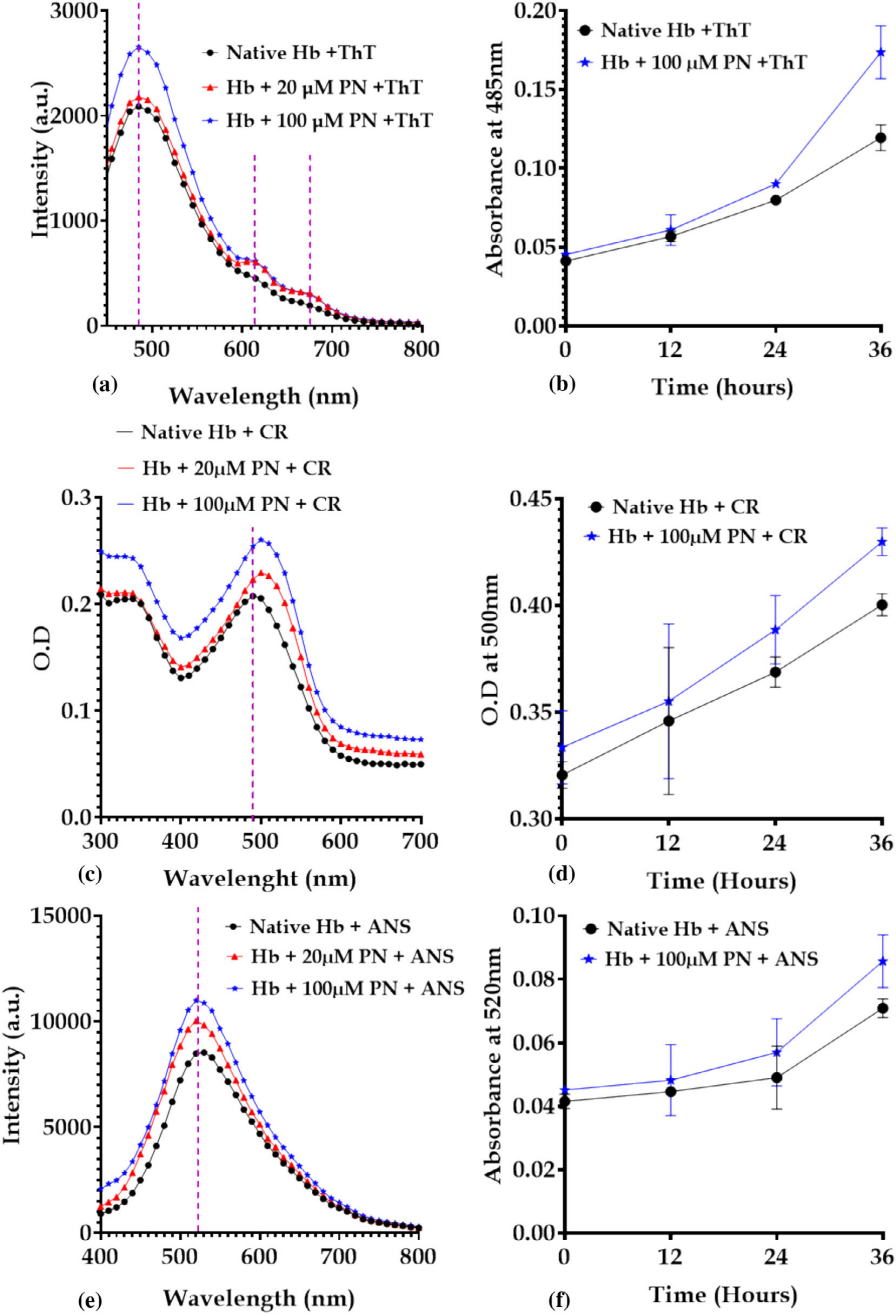
Amyloid‐sensitive dye assays of native Hb and PN‐treated Hb. (a) ThT fluorescence spectra of native Hb and PN‐treated Hb recorded in the spectral range of 450–800 nm upon excitation at 400 nm; (b) time‐dependent ThT fluorescence intensity monitored at 485 nm for native Hb and Hb treated with 100 μM PN, following incubation at 37°C over 0, 12, 24, and 36 h; (c) Congo Red absorption spectra of native Hb and PN‐treated Hb recorded in the spectral range of 300–700 nm; (d) time‐dependent Congo Red absorbance monitored at 500 nm for native Hb and Hb treated with 100 μM PN, following incubation at 37°C over 0, 12, 24, and 36 h; (e) ANS fluorescence spectra of native Hb and PN‐treated Hb recorded in the spectral range of 400–800 nm upon excitation at 350 nm; (f) time‐dependent ANS fluorescence intensity monitored at 520 nm for native Hb and Hb treated with 100 μM PN, following incubation at 37°C over 0, 12, 24, and 36 h. All measurements were performed in triplicate (*N* = 3); data are presented as mean ± SD.

The Thioflavin T (ThT) fluorescence spectra of native and PN‐treated Hb (Figure 2a), upon excitation at 400 nm, native Hb produced a broad emission band with a maximum around 485–490 nm, reflecting weak baseline ThT binding to the non‐aggregated protein. Treatment with 20 μM PN resulted in a modest increase in fluorescence intensity near 485–490 nm, while treatment with 100 μM PN produced a further concentration‐dependent increase in ThT fluorescence, without an appreciable shift in the emission maximum. The progressive increase in ThT fluorescence with increasing PN concentration suggests concentration‐dependent formation of ThT‐binding aggregates. To examine whether incubation at 37°C promoted further aggregate growth, ThT fluorescence intensity at 485 nm was monitored for native Hb and 100 μM PN‐treated Hb at 0, 12, 24, and 36 h in sealed low‐protein‐binding polypropylene microcentrifuge tubes (Figure 2b). At time zero, ThT fluorescence was low and comparable between the two conditions. Both native Hb and PN‐treated Hb showed a gradual increase in fluorescence over the 36 h incubation period; however, PN‐treated Hb exhibited consistently higher ThT intensity at each time point, indicating greater ThT‐positive aggregate formation following PN treatment during prolonged incubation.

### 2.6 Congo red dye

The Congo Red (CR) absorption spectra of native and PN‐treated Hb in the presence of CR are shown in (Figure 2c), where native Hb showed a primary absorption band around 495 nm with low overall absorbance. Treatment with 20 μM PN led to an increase in absorbance and a slight red shift of the primary absorption band, while treatment with 100 μM PN resulted in a further increase in absorbance accompanied by a more pronounced red shift and a visible shoulder on the longer‐wavelength side of the band. The concentration‐dependent increase in absorbance and red shift of the CR band suggested progressive formation of CR‐binding aggregates with increasing PN concentration. To assess whether prolonged incubation at 37°C promoted further aggregate formation, CR absorbance at 500 nm was recorded for native Hb and 100 μM PN‐treated Hb at 0, 12, 24, and 36 h (Figure 2d). At time zero, both conditions displayed comparable baseline absorbance values. CR absorbance at 500 nm increased progressively in both samples over the 36 h incubation period; however, PN‐treated Hb showed consistently higher absorbance at every time point. The steady rise in CR absorbance over time indicated progressive accumulation of CR‐binding aggregates, with PN‐treated Hb exhibiting greater aggregate formation throughout the incubation period.

### 2.7 Surface hydrophobicity

The ANS fluorescence spectra of native and PN‐treated Hb are shown in (Figure 2e). Native Hb exhibited a broad emission band centered near 520 nm upon excitation at 350 nm, reflecting the baseline hydrophobic surface accessibility of the folded tetramer. Treatment with 20 μM PN increased ANS fluorescence intensity without an appreciable shift in the emission maximum, indicating an increase in the accessibility of surface hydrophobic sites without major conformational disruption. At 100 μM PN, fluorescence intensity increased further compared with both native and 20 μM PN‐treated Hb, suggesting a concentration‐dependent increase in hydrophobic surface exposure. To determine whether prolonged incubation at 37°C promoted further hydrophobic surface exposure, ANS fluorescence intensity at 520 nm was monitored for native Hb and 100 μM PN‐treated Hb at 0, 12, 24, and 36 h (Figure 2f). At time zero, ANS fluorescence was comparable between the two conditions. Over the 36 h incubation period, PN‐treated Hb exhibited a progressive increase in ANS fluorescence intensity that remained consistently higher than that of native Hb at each time point, with the difference becoming more pronounced at 36 h. The progressive increase in ANS fluorescence indicated greater hydrophobic surface exposure in PN‐treated Hb during prolonged incubation.

### 2.8 Fourier transform infrared spectroscopy

**FIGURE 3:**
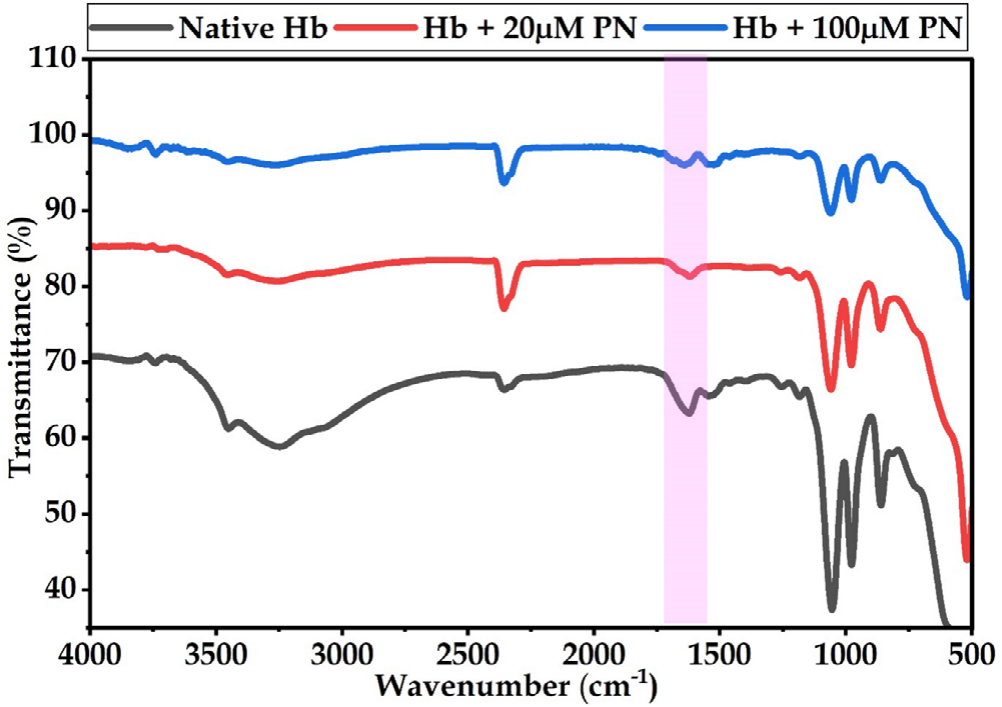
FTIR spectra of native Hb and PN‐treated Hb at 4000–500 cm^−1^.

**FIGURE 4:**
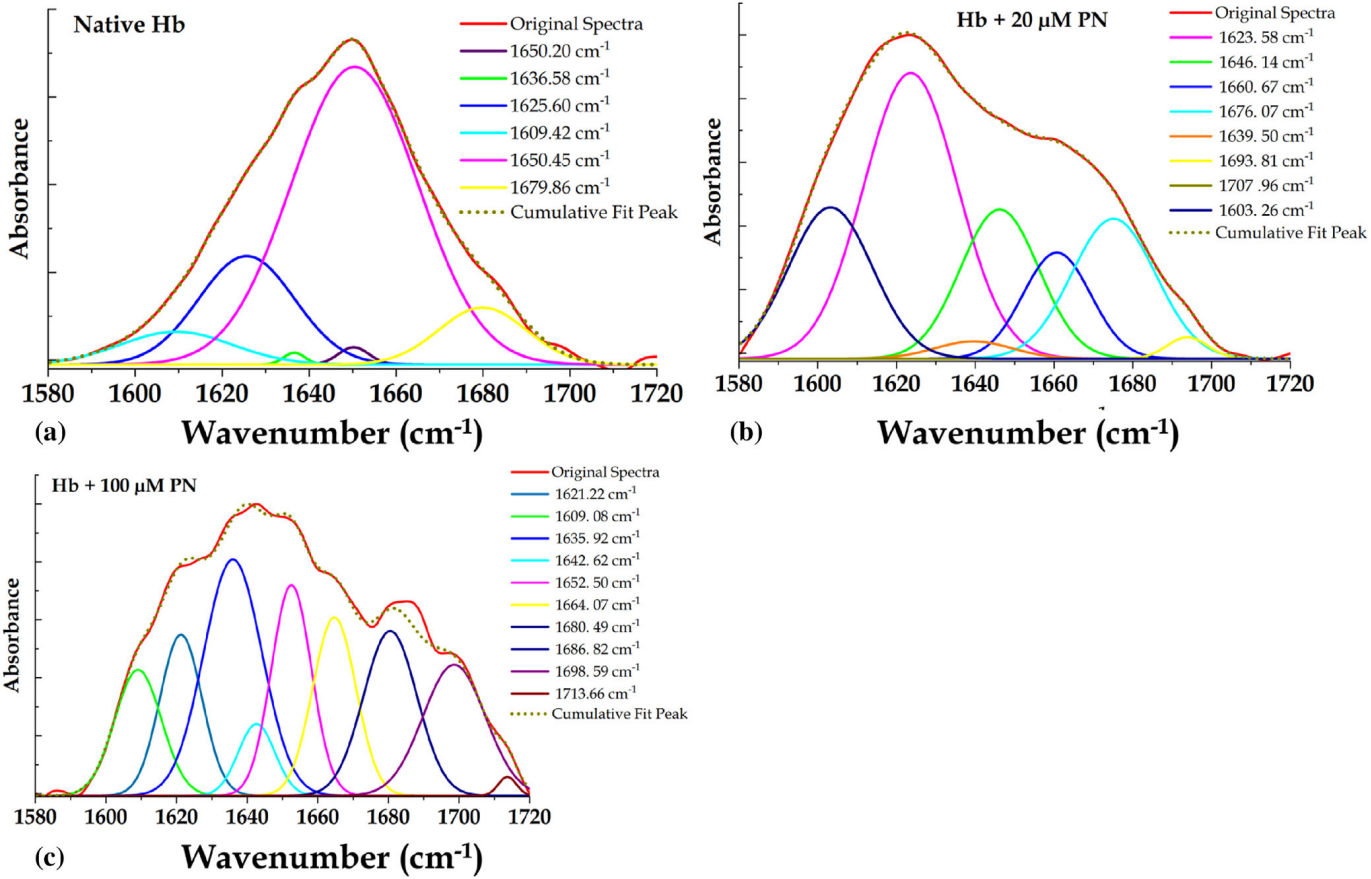
Gaussian deconvolution of the Amide I band FTIR spectra of native Hb and Hb treated with 20 and 100 μM PN.

FTIR spectroscopy was employed to examine the secondary structural alterations in native and PN‐modified Hb. The full‐range FTIR spectra (4000–500 cm^−1^) of native and PN‐treated Hb are presented in (Figure 3), where the characteristic Amide I and Amide II bands are visible in all samples. To resolve the individual secondary structural components of the Amide I band (1580–1720 cm^−1^), Gaussian curve fitting was performed using OriginPro 2026, and the deconvolved spectra are shown in (Figure 4). The relative contributions of *α*‐helix, *β*‐sheet, *β*‐turn, and random coil structures, calculated from the integrated peak areas, are summarized in Table 1. Native Hb exhibited a predominantly *α*‐helical conformation, with 66.41% *α*‐helix, 24.89% *β*‐sheet, 8.70% *β*‐turns, and no detectable random coil. Treatment with 20 μM PN reduced the *α*‐helix content to 12.06%, accompanied by increases in *β*‐sheet (48.61%), *β*‐turns (19.72%), and random coil (19.61%). At 100 μM PN, the *α*‐helix content decreased further to 8.81%, whereas *β*‐sheet content increased to 71.18%, with *β*‐turns and random coil accounting for 17.27% and 2.74%, respectively. These results demonstrate a concentration‐dependent decrease in *α*‐helical content with a corresponding increase in *β*‐sheet structures following PN treatment.

**TABLE 1:** Secondary structure content (%) of native Hb and peroxynitrite‐treated Hb determined from FTIR Amide I band deconvolution.

Sample	*α*‐helix (%)	*β*‐sheet (%)	*β*‐turn (%)	Random coil (%)
Native Hb	66.41	24.89	8.70	0.00
Hb + 20 μM PN	12.06	48.61	19.72	19.61
Hb + 100 μM PN	8.81	71.18	17.27	2.74

### 2.9 Scanning electron microscopy

**FIGURE 5:**
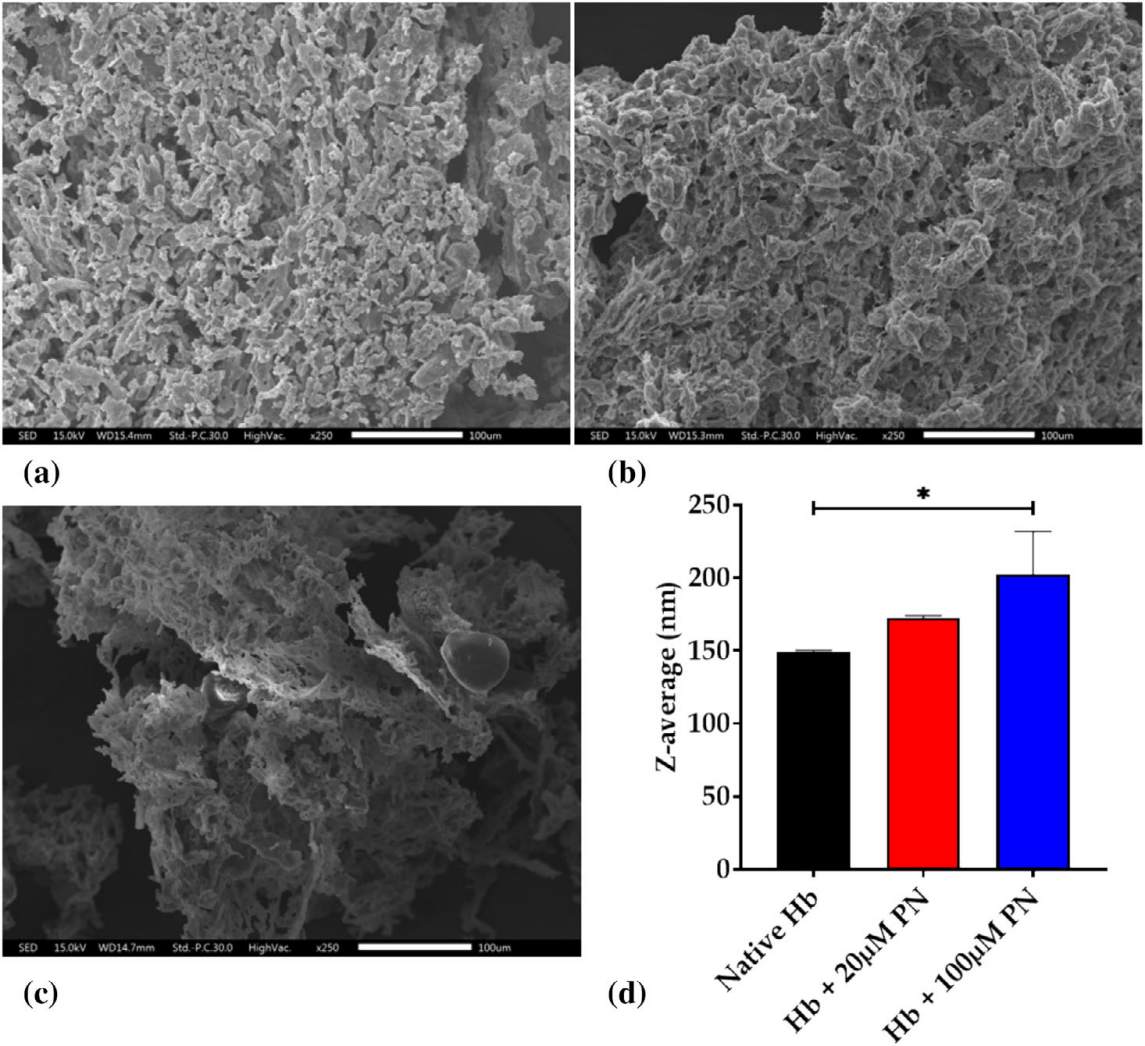
Scanning electron microscopy (SEM) and dynamic light scattering analysis (DLS) findings of native Hb and PN‐treated Hb. (a) Native Hb; (b) PN (20 μM) treated Hb; (c) PN (100 μM) treated Hb; and (d) The Z‐average hydrodynamic diameter of native Hb and PN‐modified Hb was determined by DLS. Data represent mean ± standard deviation of three independent measurements (*n* = 3). Statistical significance was assessed using Welch and Brown‐Forsythe versions of one‐way ANOVA; significance levels are denoted as *p< 0.05. The SEM images captured at ×250 and 100 μm scalebar for native and PN‐modified Hb to facilitate direct morphological comparison.

Scanning electron microscopy (SEM) was employed to evaluate the morphological alterations in Hb following PN treatment (Figure 5). Native Hb (Figure 5a) exhibited a relatively dispersed and irregular surface morphology without dense interconnected networks or large aggregate assemblies, consistent with preserved structural integrity under the control conditions. In contrast, treatment with 20 μM PN (Figure 5b) resulted in increased surface roughness, localized clustering, and partial compaction of the protein matrix. These morphological changes became more pronounced at 100 μM PN (Figure 5c), where densely packed, irregular, interconnected aggregate‐like structures were observed, indicating a concentration‐dependent increase in aggregate formation following PN treatment.

### 2.10 Dynamic light scattering (DLS) analysis

The Z‐average hydrodynamic diameter, polydispersity index (PDI), and zeta potential of native Hb and PN‐treated Hb were determined by DLS (Table S2, Supporting Information; raw instrument outputs in Figures S2, S3). The Z‐average hydrodynamic diameter of native Hb (Figure 5d) was approximately 148–150 nm, with PDI values of 0.28–0.35 and zeta potentials of −11 to −13 mV. Treatment with 20 μM PN increased the hydrodynamic diameter to 171–174 nm, with PDI values of 0.29–0.38 and zeta potentials of −5 to −6 mV. At 100 μM PN, the hydrodynamic diameter increased further to 178–235 nm, with PDI values of 0.40–0.47 and zeta potentials of −1.9 to −2.4 mV. The corresponding intensity‐weighted size distributions (Figures S2 and S3) showed the appearance of larger particle populations at higher PN concentrations while retaining species within the native size range. A progressive reduction in the magnitude of the negative zeta potential was also observed with increasing PN concentration. The mean Z‐average hydrodynamic diameters (*n* = 3) are presented (Figure 5d), with Welch and Brown–Forsythe one‐way ANOVA indicating significant differences among the groups (*p< 0.05).

### 2.11 Fluorescence microscopy

**FIGURE 6:**
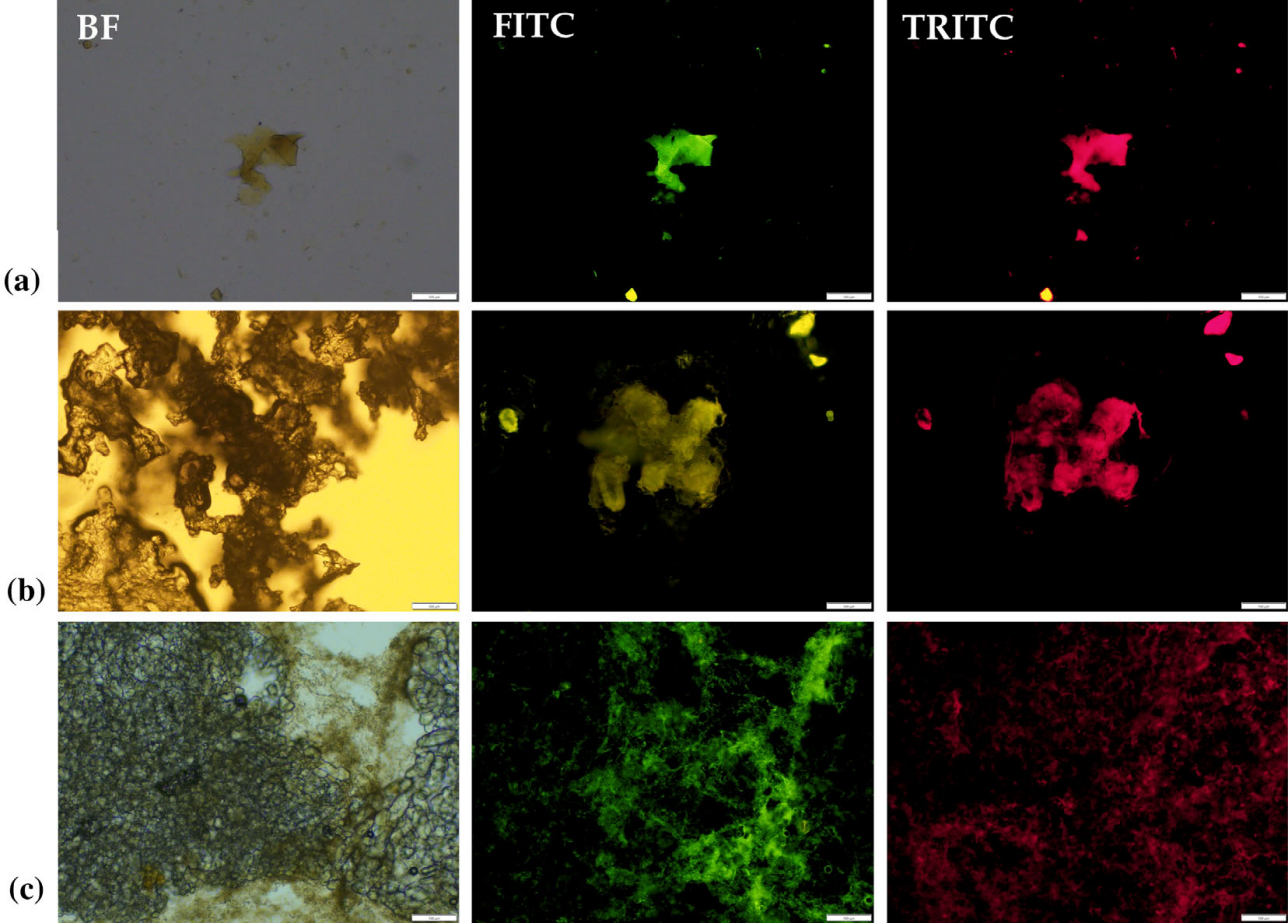
Fluorescence microscopy images of native Hb and PN‐treated Hb. (a) Native Hb; (b) PN (20 μM) treated Hb; and (c) PN (100 μM) treated Hb. All samples were subsequently incubated with 20 μM ThT for 24 h prior to imaging. The images were captured with a 100 μm scalebar for native and PN‐modified Hb to facilitate direct morphological comparison.

Fluorescence microscopy of Hb incubated with PN revealed concentration‐dependent changes in aggregate distribution and ThT‐associated fluorescence (Figure 6a–c). Native Hb showed faint, sparse ThT fluorescence in the green channel and negligible red emission, indicating minimal ThT‐positive aggregates under the imaging conditions. At 20 μM PN, Hb exhibited brighter ThT fluorescence with discrete, clustered assemblies of varying size and shape, indicating the formation of PN‐induced ThT‐positive aggregates. At 100 μM PN, the ThT‐positive aggregates appeared larger, denser, and more interconnected, forming network‐like structures. Overall, the fluorescence images demonstrated a PN concentration‐dependent increase in ThT‐positive aggregate formation.

### 2.12 Atomic force microscopy

**FIGURE 7:**
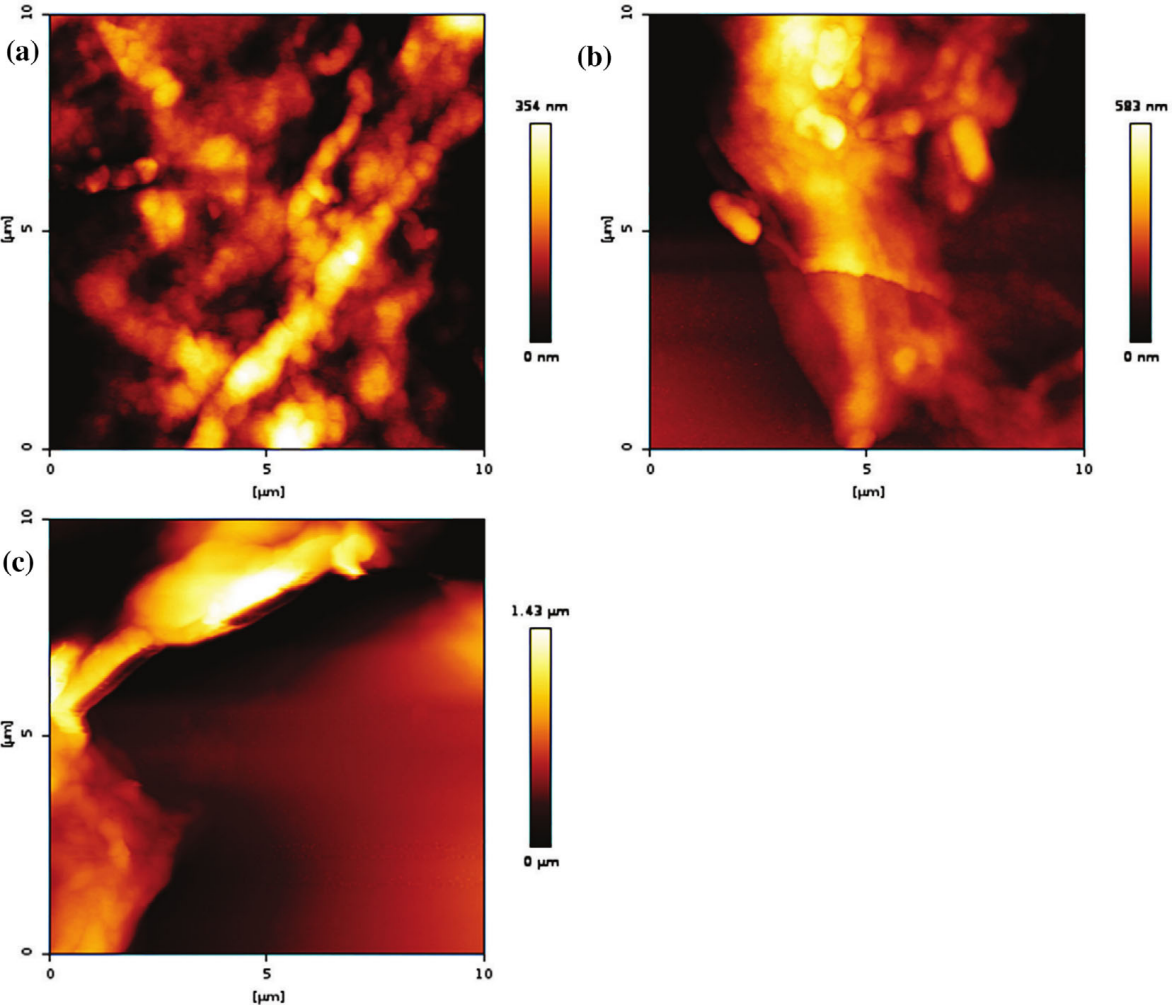
Atomic force microscopy (AFM) height topography images of native Hb and PN‐treated Hb. (a) Native Hb; (b) PN (20 μM) treated Hb; and (c) PN (100 μM) treated Hb.

**FIGURE 8:**
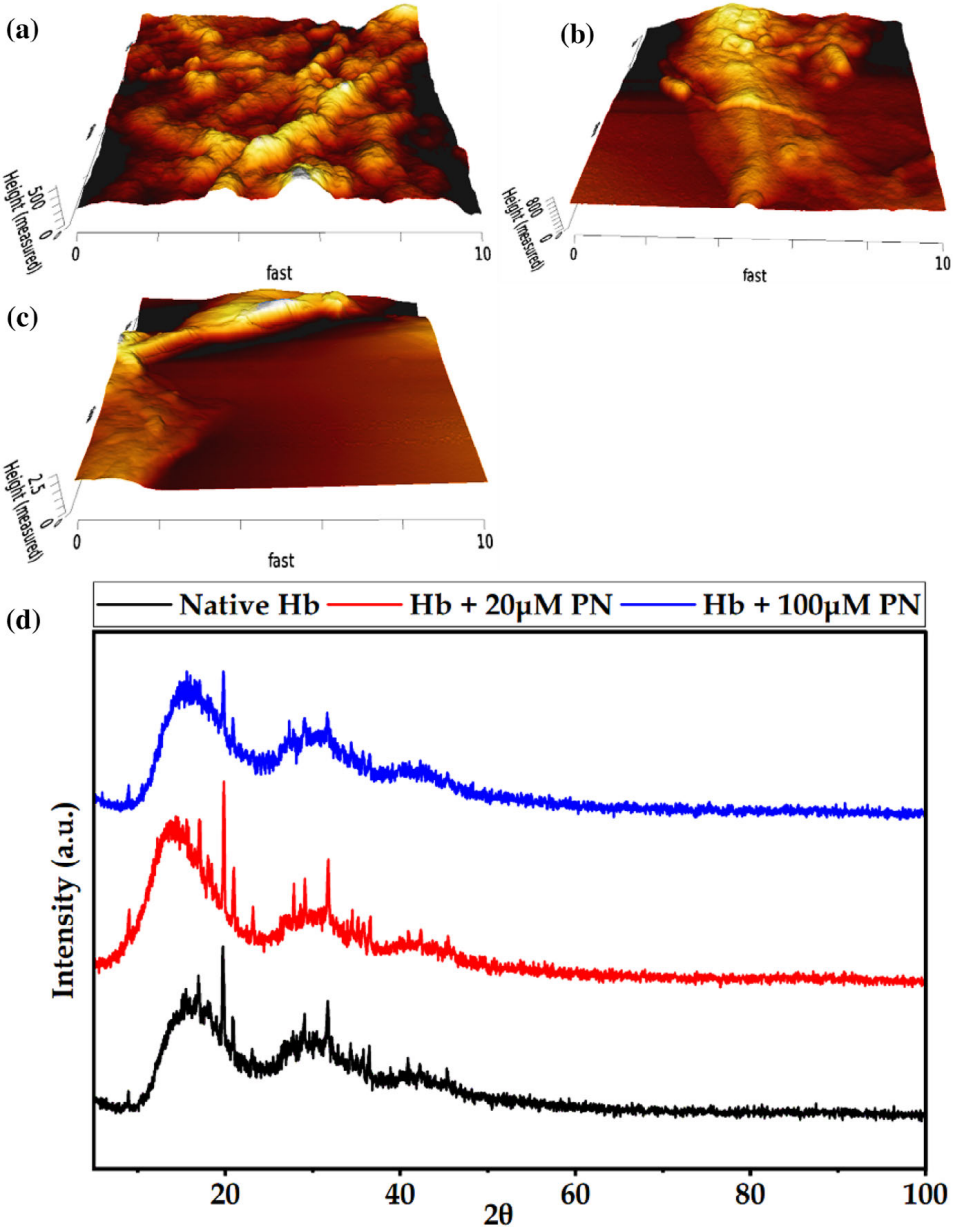
Atomic force microscopy (AFM) 3D measured Height trace and XRPD spectra of native Hb and PN‐treated Hb. (a) Native Hb; (b) PN (20 μM) treated Hb; (c) PN (100 μM) treated Hb; and (d) XRPD spectra of native Hb and PN‐treated Hb at 2*θ* (5–100).

Atomic force microscopy (AFM) height topography images (Figure 7a–c) and corresponding 3D height profiles (Figure 8a–c) revealed clear, concentration‐dependent morphological changes in Hb following PN treatment. Native Hb displayed relatively uniform surface features with moderate nanoscale undulations, with average (Ra) and RMS (Rq) roughness values of 61.97 nm and 80.36 nm, respectively (Table S3, Supporting Information), indicating a relatively homogeneous surface organization. Treatment with 20 μM PN increased surface irregularity and produced larger protrusive domains, accompanied by higher roughness values (Ra = 98.52 nm; Rq = 132.5 nm). At 100 μM PN, AFM images revealed pronounced aggregate clusters and greater height heterogeneity in both 2D and 3D views, with roughness values increasing to Ra = 238.2 nm and Rq = 325.8 nm; the corresponding roughness maps are provided in Figures S4–S6. Overall, AFM analysis demonstrated a concentration‐dependent increase in surface roughness and aggregate formation following PN treatment.

### 2.13 X‐ray powder diffraction

X‐ray powder diffraction (XRPD) patterns of native Hb and PN‐treated Hb were recorded over a 2θ range of 5–100° (Figure 8d). Native Hb displayed a broad scattering background with several superimposed maxima. A relatively intense composite feature was observed in the low‐angle region, with a broad band centered near 2*θ*≈18–22° and multiple sharper peaks superimposed on this envelope. Additional lower‐intensity peaks were present in the mid‐angle region, particularly between 2*θ*≈30–40°. Treatment with 20 μM PN retained the overall diffraction profile but reduced the prominence of the low‐angle feature and slightly decreased the prominence of peaks in the 2*θ*≈30–40° region. At 100 μM PN, the diffraction pattern became more diffuse, with only weak residual features remaining in the 2*θ*≈18–22° and 30–40° regions and no new sharp reflections observed at higher angles.

## 3 DISCUSSION

PN, formed by the rapid reaction of nitric oxide with superoxide, is a potent oxidant implicated in cardiovascular and neurodegenerative diseases through its ability to induce protein oxidation and nitration (Pacher et al., 2007). Hemoglobin (Hb) functions both as an efficient scavenger of PN and as a target of nitroxidative modification (Herold & Fago, 2005; Kalinga, 2006). Therefore, understanding how controlled PN exposure alters Hb structure and promotes aggregation provides insight into the molecular consequences of nitroxidative stress under physiologically relevant conditions (Romero et al., 2003). In the present study, intrinsic fluorescence, UV–visible spectroscopy, oxidative assays, and complementary structural analyses were employed to investigate the effects of PN on Hb. The intrinsic fluorescence data (Ex 285 nm) demonstrate a concentration‐dependent decrease in Trp/Tyr emission intensity without substantial spectral shifts. This behavior is consistent with previous studies showing that deep‐UV autofluorescence of Hb is highly sensitive to local structural perturbations around aromatic residues, whereas major wavelength shifts generally occur only after extensive unfolding or formation of fluorescent adducts (Mukunda et al., 2022, 2024). Accordingly, the gradual fluorescence quenching observed here suggests progressive oxidative modification near aromatic residues and the heme environment while indicating that the overall globin fold remains largely preserved.

This interpretation is supported by previous mechanistic studies describing the reaction of PN with Hb. PN rapidly reacts with oxyhemoglobin through heme‐centered pathways to generate methemoglobin (Pietraforte et al., 2003), nitrate, hydrogen peroxide, and transient ferryl intermediates (Romero et al., 2003), while only limited tyrosine nitration occurs under sub‐stoichiometric PN:Hb ratios (Herold & Fago, 2005; Kalinga, 2006). In addition, PN‐induced modulation of Hb peroxidase‐like activity has been attributed to subtle structural rearrangements rather than extensive protein denaturation (Xiao et al., 2009). Consistent with these reports, the smooth concentration‐dependent fluorescence quenching observed in the present study is most compatible with localized oxidative and nitrative modifications surrounding the heme pocket and aromatic residues rather than large‐scale disruption of the tetrameric structure. From a physiological perspective, these findings further support the established role of Hb as an effective scavenger of reactive nitrogen species. Under conditions where PN is present in the nanomolar‐to‐micromolar range, Hb predominantly consumes PN through heme‐centered oxidation, thereby limiting extensive nitrative damage to the globin chain (Herold & Fago, 2005; Kalinga, 2006; Pietraforte et al., 2003; Romero et al., 2003; Xiao et al., 2009). The fluorescence response observed here is therefore consistent with controlled oxidative conversion of the heme center accompanied by relatively modest perturbations of neighboring Trp and Tyr residues (Herold & Fago, 2005; Mukunda et al., 2022, 2024).

The UV–visible absorption spectra complement the fluorescence findings by directly probing changes in the heme electronic environment. Increasing PN concentration did not produce new absorption maxima or appreciable wavelength shifts but resulted in a gradual increase in absorbance intensity and spectral broadening in the UV and visible regions. Similar spectral behavior has been reported during PN‐mediated oxidation of Hb, where conversion to methemoglobin and ferryl intermediates produces moderate absorbance changes without major disruption of the globin–heme architecture (Romero et al., 2003); (Herold & Fago, 2005; Kalinga, 2006) and (Pietraforte et al., 2003). Comparable deep‐UV spectroscopic studies have likewise shown that moderate chemical modification of Hb increases absorbance while simultaneously quenching intrinsic fluorescence without evidence of extensive unfolding (Mukunda et al., 2022, 2024). Collectively, the fluorescence and UV–visible results indicate controlled heme‐centered oxidation accompanied by limited perturbation of the surrounding aromatic environment while preserving the overall structural integrity of Hb.

The oxidative fluorescence measurements obtained at 325 nm complement the intrinsic fluorescence and UV–visible analyses by probing tyrosine‐centered oxidative chemistry associated with PN‐induced protein modification (Prabhu et al., 2018; Shah et al., 2026). Previous studies have consistently shown that PN promotes oxidation of Tyr and Trp residues, dityrosine formation, and protein aggregation in several globular proteins. In human IgG and serum albumin, PN treatment induces intrinsic fluorescence quenching together with the appearance of 325 nm‐excited emission attributed primarily to dityrosine and related oxidative products, accompanied by aggregation detected by complementary biophysical techniques (Arfat et al., 2016); (Arif et al., 2017). Additional studies have demonstrated that blue‐visible fluorescence from protein aggregates may arise from multiple oxidized chromophores rather than a single fluorophore, suggesting that aggregate‐associated emission reflects chemically heterogeneous species (Radomska & Wolszczak, 2022). For Hb, oxidative stress has similarly been shown to generate stable dityrosine and other tyrosine oxidation products, while mechanistic studies indicate that PN‐mediated ferryl intermediates and globin‐centered radicals promote oxidative crosslinking with relatively limited tyrosine nitration under physiological PN:Hb ratios (Giulivi & Davies, 1993); (Romero et al., 2003) and (Su & Groves, 2010). Accordingly, the red‐shifted fluorescence bands observed in the present study are consistent with PN‐induced oxidation and aggregation of Hb involving multiple oxidized and crosslinked species rather than a single emissive product.

The DNPH assay further confirmed progressive oxidative modification of Hb through concentration‐dependent carbonyl formation. Similar increases in protein carbonyls following PN exposure have been reported for human serum albumin and IgG, where carbonyl accumulation occurred alongside nitration, dityrosine formation, and aggregation (Arfat et al., 2016) and (Arif et al., 2017). Comparable dose‐dependent increases in carbonyl content have also been observed in oxidized food proteins, supporting carbonyl formation as a general marker of irreversible oxidative damage (HU et al., 2023). The graded increase in DNPH‐reactive carbonyls observed here, therefore, complements the fluorescence and UV–visible data and confirms effective oxidative modification of Hb by PN.

To determine whether these oxidative modifications promote higher‐order structural organization, aggregation was examined using ThT, CR, and ANS. The moderate increase in ThT fluorescence with increasing PN concentration indicates the formation of *β*‐sheet‐associated aggregates but does not suggest the extensive fibrillation typically observed in strongly amyloidogenic proteins. Previous studies have similarly shown that oxidative or glycative modification of Hb, albumin, and other globular proteins enhances ThT fluorescence through partial unfolding, aromatic residue modification (Chikkanayakanahalli Mukunda et al., 2025; Mukunda et al., 2022, 2024) and formation of mixed oligomeric or *β*‐sheet‐enriched assemblies rather than mature fibrils (Xiao et al., 2024). Consistent with these reports, the modest ThT enhancement observed here suggests that PN promotes early aggregation while preserving much of the native Hb architecture. Time‐dependent ThT measurements further showed progressive increases in fluorescence during incubation at 37°C for both native and PN‐treated Hb, with PN‐treated samples consistently exhibiting higher intensities. These findings suggest that oxidative modification increases the susceptibility of Hb to subsequent thermally driven aggregation, resulting in accelerated aggregate accumulation over time.

The CR assay supported this interpretation. Whereas highly ordered amyloid fibrils typically produce pronounced CR spectral shifts and increased absorbance, oxidative aggregation of globular proteins often results in only modest spectral changes associated with mixed amorphous and partially ordered assemblies (Lyu et al., 2020; Mahdavimehr et al., 2017). The concentration‐dependent increase in CR absorbance and the limited red shift observed for PN‐treated Hb therefore suggest partial *β*‐sheet organization without formation of long, highly ordered amyloid fibrils. Likewise, the progressive increase in CR binding during incubation indicates gradual accumulation of aggregation‐prone species, which is enhanced by prior PN‐induced oxidation.

ANS fluorescence provided complementary information regarding hydrophobic surface exposure. The concentration‐dependent increase in ANS intensity indicates partial exposure of hydrophobic regions following PN treatment, consistent with limited conformational loosening rather than extensive unfolding. Similar increases in ANS fluorescence have been reported during oxidative modification of fibrinogen, whereas PN‐treated HSA exhibited reduced ANS binding because oxidation promoted burial rather than exposure of hydrophobic regions, illustrating that ANS responses are protein dependent (Arif et al., 2017) and (Farhana et al., 2024; Figueroa et al., 2022). Comparable observations have also been reported for glycatively modified HSA, where changes in ANS fluorescence reflected localized rearrangements of hydrophobic domains rather than global denaturation (Mukunda et al., 2022, 2024). In the present study, the absence of appreciable shifts in the ANS emission maximum further supports the conclusion that PN induces only limited structural loosening while maintaining the overall globular architecture of Hb. Time‐dependent ANS measurements similarly demonstrated progressive increases in hydrophobic surface accessibility during incubation, with PN‐treated Hb exhibiting consistently greater fluorescence than native Hb throughout the experiment.

Together with the ThT and CR results, these findings indicate that PN‐induced oxidation facilitates gradual structural remodeling and aggregation while remaining distinct from the extensive conformational changes associated with classical amyloid fibrillation. Because all incubation experiments were performed under static conditions in sealed, low‐protein‐binding amber polypropylene tubes without agitation, the contribution of container‐induced aggregation is expected to be minimal. Previous studies have shown that significant surface‐induced protein aggregation generally requires the combined effects of air–liquid interfaces, solid surfaces, and agitation, whereas static incubation markedly reduces these effects (Schvartz et al., 2023). Consequently, the time‐dependent spectroscopic changes observed here are most reasonably attributed to PN‐induced oxidative modification and subsequent incubation‐dependent structural evolution rather than artifacts arising from the experimental container.

FTIR analysis of the amide I region demonstrated that PN induces concentration‐dependent secondary structural remodeling of Hb, characterized by a reduction in *α*‐helical content and a corresponding increase in *β*‐sheet structures. Native Hb is predominantly *α*‐helical, and the secondary‐structure composition observed in the present study agrees with previous spectroscopic characterizations (Mukhopadhyay et al., 2022). Similar *α*‐helix‐to‐*β*‐sheet transitions have been reported following oxidative stress in Hb and other globular proteins, including ionizing radiation‐induced oxidation of Hb, metal‐catalyzed oxidation of human serum albumin, heat‐induced aggregation of bovine serum albumin, and PN‐induced structural modification of IgG (Arfat et al., 2016); (Brahma et al., 2024); (Hinge et al., 2024) and (Mukhopadhyay et al., 2022). Likewise, ATR‐FTIR studies of PN‐treated amyloid‐*β* demonstrated enhanced *β*‐sheet stabilization following nitrotyrosination (Guivernau et al., 2016). Collectively, these findings support the interpretation that PN promotes partial unfolding and *β*‐sheet enrichment in Hb while increasing structural heterogeneity, consistent with the spectroscopic evidence for oxidative modification observed in the present study.

The microscopic analyses provide complementary morphological evidence for these structural changes. SEM revealed a progressive transition from dispersed native Hb particles to increasingly compact and clustered aggregates with increasing PN concentration. Similar oxidative crosslinking‐induced aggregation has previously been reported for PN‐treated human serum albumin and chemically modified hemoproteins (Mukunda et al., 2024) and (Arif et al., 2017). Because SEM cannot identify the underlying chemical modifications, these observations are best interpreted as morphological correlates of the oxidative and structural changes demonstrated by the spectroscopic analyses rather than direct evidence of specific crosslinking mechanisms. The DLS measurements further showed a concentration‐dependent increase in the hydrodynamic diameter of Hb together with changes in polydispersity and zeta potential, indicating the formation of progressively larger particle populations following PN treatment. Similar increases in particle size have been reported for chemically modified hemoglobins and oxidatively stressed proteins, where DLS sensitively detects changes in aggregation state accompanying structural modification (Faggiano et al., 2010); (Feng et al., 2022); (Santiago et al., 2008); and (HU et al., 2023). The accompanying reduction in the magnitude of the negative zeta potential suggests altered surface charge following oxidation, which is qualitatively consistent with increased intermolecular association. Nevertheless, DLS does not resolve detailed aggregate architecture, and these data should therefore be interpreted as evidence of solution‐phase particle growth rather than specific aggregation mechanisms.

Fluorescence microscopy further supported the aggregation pathway indicated by the dye‐binding assays. Native Hb exhibited weak ThT fluorescence, whereas PN‐treated samples displayed progressively brighter and more heterogeneous fluorescent clusters with increasing oxidant concentration. Similar observations have been reported during oxidative and glycative modification of proteins, where increased ThT fluorescence reflects heterogeneous populations of oligomeric, amorphous, and partially *β*‐sheet‐enriched aggregates rather than exclusively mature amyloid fibrils (Basha, Mukunda, et al., 2026; Basha, Pranavi, et al., 2026); (Toprakcioglu et al., 2019) and (Mukunda et al., 2024). The predominance of compact, irregular aggregates instead of elongated fibrillar structures, therefore, suggests that PN promotes structurally heterogeneous aggregate formation under the present experimental conditions.

AFM provided complementary nanoscale characterization of these aggregates. Progressive increases in surface roughness together with the appearance of clustered protrusive structures indicate concentration‐dependent nanoscale remodeling of Hb following PN exposure. Comparable increases in topographical heterogeneity have been associated with oxidative protein reorganization and aggregation in erythrocyte systems and thermally stressed proteins (Starodubtseva et al., 2007) and (Basha, Pranavi, et al., 2026). Although AFM cannot identify specific chemical crosslinks or secondary‐structure motifs, the observed changes support the conclusion that PN induces progressive structural reorganization that is consistent with the spectroscopic and microscopic findings. XRPD analysis provided complementary information regarding the long‐range structural organization of PN‐treated Hb. The broad halo‐like diffraction patterns observed at all PN concentrations, without the appearance of new sharp Bragg reflections, indicate that PN‐induced aggregation does not generate highly ordered crystalline or amyloid‐like assemblies under the present conditions. Similar diffuse diffraction profiles have been reported for thermally aggregated globular proteins and stored protein powders, whereas highly ordered hemoglobin polymers and amyloid‐like aggregates produce distinct crystalline reflections that were absent in the present study (Banerjee & Chakraborti, 2016); (Basha, Pranavi, et al., 2026); (Mitsudome, 2024) and (Magdoff‐Fairchild & Chiu, 1979).

**FIGURE 9:**
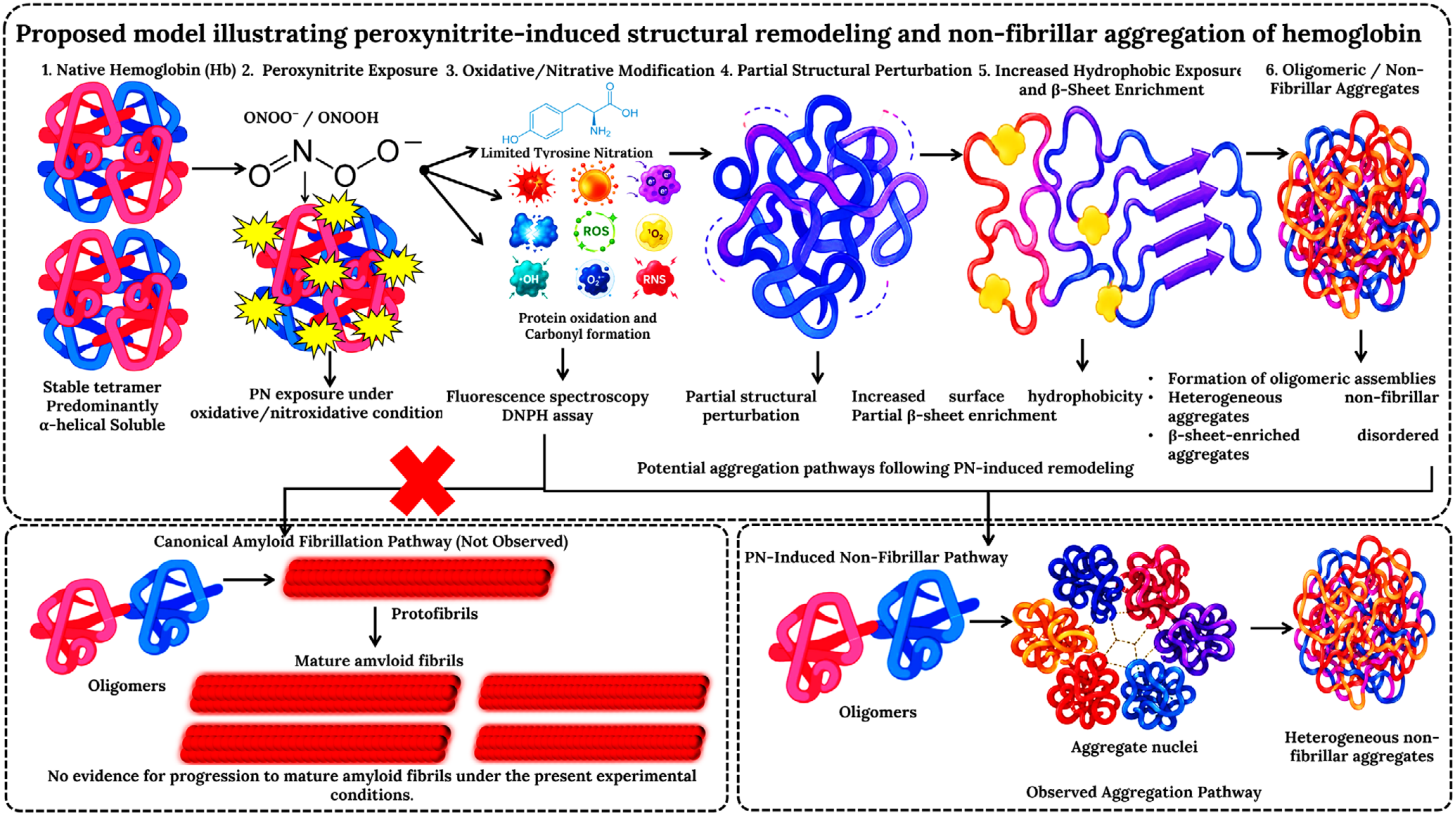
Proposed model illustrating peroxynitrite‐induced structural remodeling and non‐fibrillar aggregation of hemoglobin. Native hemoglobin undergoes oxidative and limited nitrative modification following exposure to peroxynitrite (PN) under oxidative/nitroxidative conditions, resulting in protein oxidation, carbonyl formation, and partial structural perturbation. These structural changes are accompanied by increased surface hydrophobicity and partial *β*‐sheet enrichment, promoting the formation of oligomeric assemblies that subsequently develop into heterogeneous non‐fibrillar aggregates. Although *β*‐sheet‐enriched aggregates are formed, no evidence for progression toward the canonical amyloid fibrillation pathway (oligomers to protofibrils to mature amyloid fibrils) was not observed under the present experimental conditions, consistent with the absence of long, highly ordered fibrillar structures in XRPD, AFM, and fluorescence analyses. The canonical fibrillation pathway is therefore shown for comparison only, whereas the non‐fibrillar pathway represents the experimentally observed aggregation route. This model integrates the experimental findings of the present study together with established concepts in protein aggregation biology; in particular, studies on nitrotyrosinated A*β* have shown that peroxynitrite‐dependent Tyr10 nitration can stabilize oligomers and divert aggregation toward non‐fibrillar, amorphous assemblies rather than mature fibrils (Basile et al., 2024; Guivernau et al., 2016).

These findings therefore support the conclusion that PN favors the formation of structurally heterogeneous, poorly ordered aggregates rather than long‐range crystalline architectures. The absence of highly ordered fibrillar assemblies is also qualitatively consistent with previous studies showing that PN‐mediated nitrotyrosination can alter protein aggregation pathways. In amyloid‐*β*, nitrotyrosination has been shown to stabilize soluble oligomeric or amorphous aggregates while limiting the formation of mature fibrils (Basile et al., 2024) and (Guivernau et al., 2016). Although Hb differs fundamentally from amyloid‐*β* and the present study does not identify residue‐specific PN modifications, the combined spectroscopic, structural, and microscopic data consistently indicate that PN promotes partial unfolding, *β*‐sheet enrichment, and formation of oligomeric or amorphous aggregates without generating long, highly ordered amyloid fibrils. Overall, the complementary findings obtained from intrinsic and oxidative fluorescence, UV–visible spectroscopy, DNPH carbonyl analysis, ThT, Congo Red, ANS, FTIR, DLS, SEM, fluorescence microscopy, AFM, and XRPD support a coherent model in which PN initially induces heme‐centered oxidation accompanied by localized modification of aromatic residues. These early oxidative events promote progressive secondary structural remodeling, increased hydrophobic exposure, and intermolecular association, ultimately leading to the formation of heterogeneous *β*‐sheet‐enriched oligomeric and amorphous Hb aggregates, as proposed in the schematic pathway in (Figure 9). Under the experimental conditions employed, however, these structural changes remain distinct from the highly ordered fibrillar assembly characteristic of classical amyloid formation, suggesting that additional biochemical or physiological factors may be required to drive Hb toward a fully amyloidogenic state.

## 4 CONCLUSION, LIMITATIONS AND OUTLOOK

This study demonstrates that PN induces concentration‐dependent structural remodeling of hemoglobin, leading to heterogeneous oligomeric and amorphous aggregation rather than canonical amyloid fibril formation. At low oxidant concentrations, hemoglobin efficiently scavenges PN through heme‐centered reactions with minimal structural perturbation. Increasing PN exposure, however, exceeded this protective capacity and was associated with progressive protein carbonylation, substantial loss of *α*‐helical structure (66.41%–8.81%), increased *β*‐sheet content (71.18%), altered surface charge, and formation of structurally heterogeneous aggregates without evidence of long‐range crystalline order. Time‐dependent experiments further indicated that prior PN modification increased the susceptibility of hemoglobin to thermally driven aggregation at physiological temperature. Collectively, these findings provide a mechanistic framework for understanding peroxynitrite‐induced hemoglobin remodeling and distinguish nitroxidative protein aggregation from classical amyloid fibrillation.

This study has several limitations. Experiments were performed using purified hemoglobin in a simplified buffer system and therefore do not account for endogenous erythrocyte antioxidant defenses, including glutathione, catalase, and peroxiredoxin‐2, which are expected to influence susceptibility to nitroxidative damage. Although the spectroscopic and morphological analyses demonstrated structural remodeling, they do not identify residue‐specific oxidative modifications; therefore, LC–MS/MS‐based mapping of nitrotyrosine, dityrosine, and carbonylated residues remains necessary. Likewise, DLS measurements provide population‐average particle size and zeta potential without distinguishing covalent crosslinking from non‐covalent association, while ThT and Congo Red assays indicate β‐sheet enrichment but cannot independently confirm classical amyloid architecture. Finally, functional consequences, including oxygen‐binding affinity, cooperativity, and heme‐release kinetics, were not investigated.

Future studies should validate these findings in intact erythrocytes and physiologically relevant environments to determine how endogenous antioxidant systems influence hemoglobin stability under nitroxidative stress. Integrating LC–MS/MS proteomics with oxygen‐binding and kinetic analyses will further establish the relationship between residue‐specific oxidation, structural remodeling, and functional impairment. In addition, investigating the molecular basis for the absence of canonical amyloid fibril formation and determining whether PN‐modified hemoglobin aggregates contribute to oxidative stress or damage‐associated molecular pattern (DAMP)‐mediated signaling under hemolytic and neuroinflammatory conditions will provide further insight into their potential relevance in vascular and neurodegenerative diseases.

## 5 MATERIALS AND METHODS

### 5.1 Chemicals

All reagents and chemicals used in this study were of analytical grade and used without further purification. 2,4‐Dinitrophenylhydrazine (DNPH; Cat. No. 88639) and trichloroacetic acid (Cat. No. 2384998) were obtained from Sisco Research Laboratories Pvt. Ltd. (SRL, India). Human hemoglobin (Hb; H7379), 8‐anilino‐1‐naphthalenesulfonic acid (ANS; A1028, ≥96%), guanidine hydrochloride (G3272), Congo red (C6767), Thioflavin T (T3516), and muscovite mica sheets for AFM (AFM‐71856‐03) were purchased from Sigma‐Aldrich (St. Louis, MO, USA). PN (Item No. 81565; CAS 14042‐01‐4) was obtained from Cayman Chemical (Ann Arbor, MI, USA). Sodium bicarbonate (MB045‐500G) was purchased from HiMedia Laboratories Pvt. Ltd. (Mumbai, India). All solutions were prepared using ultrapure water, and buffer solutions were freshly prepared prior to use.

### 5.2 Modification of hemoglobin by PN

Hemoglobin (15 μM, expressed as tetramer concentration) was incubated with PN at final concentrations ranging from 5 to 100 μM, corresponding to approximately 0.3–6.7 molar equivalents relative to hemoglobin, in 10 mM phosphate buffer (pH 7.4) supplemented with 10 mM sodium bicarbonate to mimic physiological CO₂/HCO₃^−^ conditions and promote physiologically relevant peroxynitrite‐derived reactions. Reaction mixtures were incubated at 37°C for 45 min. PN stock solution (supplied in 0.3 M NaOH) was thawed on ice immediately prior to use. The concentration of each aliquot was verified spectrophotometrically at 302 nm (ε = 1670 M^−1^ cm^−1^) in 0.3 M NaOH prior to dilution, and representative concentration verification data are provided in the Supporting Information. Fresh aliquots were used for each experimental series to minimize decomposition. PN was added last to initiate the reaction, and samples were gently mixed immediately after addition. Control samples received equivalent volumes of 0.3 M NaOH vehicle. Reactions were carried out in sealed low‐protein‐binding, amber‐tinted polypropylene microcentrifuge tubes to minimize evaporation and unintended exposure to ambient oxidants. All experiments were performed immediately after sample preparation unless otherwise stated. Prior to oxidant treatment, the integrity and redox state of hemoglobin were verified by UV–visible spectroscopy through analysis of the Soret band and visible absorption peaks at 541, 577, and 630 nm. These measurements confirmed the predominance of oxyhemoglobin, minimal methemoglobin formation, and the absence of detectable heme loss or pre‐existing denaturation (Figure S1, Supporting Information). All subsequent spectroscopic, biochemical, and microscopic analyses were performed using the PN‐modified Hb samples prepared as described above. Detailed experimental protocols, instrumental settings, validation experiments, and data‐processing procedures for all analytical techniques are provided in the Supporting Information.

## AUTHOR CONTRIBUTIONS

**Shaik Basha:** Conceptualization; investigation; writing – original draft; writing – review and editing; visualization; validation; methodology; software; formal analysis; data curation; resources. **Aradhika Vijeev:** Writing – review and editing. **Krishna Kishore Mahato:** Conceptualization; investigation; funding acquisition; methodology; validation; visualization; writing – review and editing; project administration; formal analysis; software; data curation; supervision; resources. **Darshan Chikkanayakanahalli Mukunda:** Writing – review and editing.

## FUNDING INFORMATION

This study was supported by the Manipal Academy of Higher Education (MAHE), Manipal, Karnataka, India. Indian Council of Medical Research (ICMR) (Ref. EM/Dev/SG/75/0782/2023, Ref. 17x(3)/Adhoc/33/2022‐ITR), Department of Biotechnology—Boost to University Interdisciplinary Life Science Departments for Education and Research (DBT‐BUILDER) (BT/INF/22/SP43065/2021), Department of Science and Technology—“Innovation in Science Pursuit for Inspired Research (DST‐INSPIRE)” (IF:220005) Govt. of India.

## CONFLICT OF INTEREST STATEMENT

The authors declare no potential conflict of interest.

## Supporting information


**Table S1.** Carbonyl content in native and PN‐treated Hb by DNPH assay.
**Figure S1.** The absorbance of native Hb before conducting experiments. (a) Absorbance scan native Hb at 200–500 nm; (b) Absorbance of native Hb at 541 nm; (c) Absorbance of native Hb at 577 nm; and (d) Absorbance of native Hb at 630 nm.
**Table S2.** Dynamic light scattering (DLS) measurements of native hemoglobin (Hb) and PN‐treated Hb samples. The Z‐average hydrodynamic diameter, polydispersity index (PDI) and Zeta potential (mV) are shown for three independent measurements of each sample.
**Figure S2.** The Z‐average hydrodynamic diameter and polydispersity index (PDI) of native Hb, Hb with 20 μM PN and Hb with 100 μM PN were determined by dynamic light scattering (DLS) based on three independent measurements.
**Figure S3.** The Zeta potential (mV) of Native Hb, Hb with 20 μM PN and Hb with 100 μM PN was determined by dynamic light scattering (DLS) based on three independent measurements.
**Table S3.** Roughness measurements of native Hb and Hb treated with Peroxynitrite.
**Figure S4.** The roughness measurements of native Hb.
**Figure S5.** The roughness measurements of 20 μM PN‐treated Hb.
**Figure S6.** The roughness measurements of 100 μM PN‐treated Hb.

## Data Availability

The data that support the findings of this study are available in the supplementary material of this article. Additional data that support the findings of this study are available from the corresponding author upon reasonable request.
