# Supplementary material for: Peroxynitrite‐induced structural remodeling and aggregation of hemoglobin under nitroxidative stress
Source: Protein Sci. 2026 Aug 12;35(9):e70762. doi: 10.1002/pro.70762 (PMC13463319; doi:10.1002/pro.70762)
Supplement: Supplementary file 1 [file PRO-35-e70762-s001.docx]

**Peroxynitrite-Induced Structural Remodeling and Aggregation of Hemoglobin under Nitroxidative Stress**

*Shaik Basha^1,^****^†^****, Aradhika Vijeev^2,^****^†^****, Darshan Chikkanayakanahalli Mukunda^1,2^, Krishna Kishore Mahato^1,^****^*^***

1. *Department of Biophysics, Manipal School of Life Sciences, Manipal Academy of Higher Education, Manipal, India.*
2. *Manipal School of Life Sciences, Manipal Academy of Higher Education, Manipal, India.*
3. *Department of Biosciences & Bioengineering, Indian Institute of Technology Dharwad, Dharwad - 580011, Karnataka, India.*

***† Authors with equal contribution***

****Corresponding author:*** [*kkmahato@gmail.com*](mailto:kkmahato@gmail.com) & [mahato.kk@manipal.edu](mailto:mahato.kk@manipal.edu)

1. **Methods**
   1.1. **Fluorescence Spectroscopy**

Fluorescence measurements were performed to monitor structural alterations in hemoglobin (Hb) following peroxynitrite exposure. Fluorescence spectra were recorded using a QE-Pro spectrofluorometer (Ocean Optics, USA) equipped with a light-emitting diode excitation source. Samples were excited at 285 nm to monitor the fluorescence of aromatic residues, with the emission signal being dominated by tryptophan residues and highly sensitive to changes in the local protein environment. Emission spectra were collected over the range of 300–600 nm. Fluorescence measurements were performed at a hemoglobin concentration of 15 μM. Slit widths, integration time, and other acquisition parameters were kept constant throughout the study to ensure direct comparison between samples. All spectra were recorded at room temperature (20 ± 1 °C) using matched 1 cm quartz cuvettes. Background buffer spectra were acquired under identical conditions and subtracted from sample spectra. Measurements were performed in triplicate, and representative spectra are shown. Additional instrumental details are provided in our previous report (Basha et al., 2026). Based on the dose-dependent fluorescence changes observed across the 5–100 μM peroxynitrite range, samples treated with 20 μM and 100 μM peroxynitrite were selected for subsequent spectroscopic and microscopic analyses. The 20 μM condition represents early structural perturbation with limited aggregation, whereas the 100 μM condition exhibits pronounced fluorescence quenching and conformational destabilization, enabling comparative assessment of progressive oxidative remodeling.

1.2. **UV-Visible Absorption Spectroscopy**

UV–visible absorption spectroscopy was performed to assess structural transitions and aggregation-associated spectral changes in native and peroxynitrite-treated hemoglobin (Hb). Spectra were recorded using a Tecan Spark multimode microplate reader (Männedorf, Switzerland). For structural analysis, spectra were acquired over the wavelength range of 220–500 nm, while extended scans were used where necessary for evaluation of hemoglobin redox-state markers. Samples (200 μl per well) were loaded into UV-transparent flat-bottom 96-well microplates, and measurements were performed at room temperature (20 ± 1 °C). Particular attention was given to absorbance near 280 nm, reflecting the local environment of aromatic residues and tertiary structural perturbations, and to the Soret band at 415 nm, which is sensitive to heme integrity and oxidation state. Changes in absorbance intensity, spectral profile, and peak position (λmax) were monitored to evaluate concentration-dependent conformational alterations and aggregation-related effects. All measurements were performed in triplicate (N = 3), and mean values were used for subsequent analysis. Instrument settings were maintained constant throughout the study to ensure comparability between samples. Additional instrumental details are available in our previous report (Basha et al., 2026).

1.3. **Detection of Oxidative Modifications by Laser-Induced Autofluorescence**

Oxidative modifications in native and peroxynitrite-treated hemoglobin (Hb) were evaluated using laser-induced autofluorescence (LIAF) spectroscopy. Native and oxidant-treated Hb samples (15 μM) were analyzed immediately following incubation. The detailed configuration of the LIAF system has been described previously (Prabhu et al., 2018). Briefly, excitation was provided by a 325 nm He–Cd laser operating at a constant output power, with approximately 2 mW delivered at the probe tip through a fiber-optic assembly. Fluorescence emission arising from intrinsic and oxidation-induced fluorophores was collected through surrounding optical fibers and directed to a spectrometer–CCD detection system. Residual excitation light was removed using a high-efficiency optical filter prior to detection. Emission spectra were recorded over the wavelength range of 300–800 nm using a fixed integration time of 10 s. Measurements were performed in a non-contact configuration with the probe positioned approximately 3 mm from the sample surface to ensure consistent signal acquisition and to minimize thermal or mechanical perturbation. All measurements were performed in triplicate under identical instrumental conditions.

1.4. **Carbonyl Content**

The determination of protein carbonyl content in native and peroxynitrite-modified hemoglobin (Hb) was performed using the 2,4-dinitrophenylhydrazine (DNPH) assay as described previously (Arif et al., 2017). An aliquot (200 µl) of native or peroxynitrite-modified Hb was mixed with 250 µl of 10 mM DNPH prepared in 2.5 M HCl and incubated for 15 min at room temperature in the dark. Corresponding blanks were prepared by replacing the DNPH solution with HCl alone. Protein was precipitated by the addition of 125 µl of 50% (w/v) trichloroacetic acid (TCA) and incubated on ice for 15 min. The precipitated protein was collected by centrifugation at 9,000 × g for 15 min at 4 °C and washed three times with 1 ml of ice-cold ethanol: acetone (1:1, v/v) to remove excess reagent. The washed pellets were dissolved in 1 ml of 2 M guanidine hydrochloride (GdnHCl) and incubated at 37 °C for 15–20 min until complete dissolution was achieved. Absorbance was measured at 370 nm for DNPH-carbonyl hydrazones and at 280 nm for protein quantification. Carbonyl content was calculated using an extinction coefficient (ε) of 22,000 M⁻¹ cm⁻¹ and expressed as nmol carbonyl groups per mg protein. All measurements were performed in triplicate (N = 3).

1.5. **Thioflavin T**

Thioflavin T (ThT) fluorescence was used to assess the formation of β-sheet-rich, amyloid-like aggregates in native and peroxynitrite-treated hemoglobin (Hb) samples. A ThT stock solution (1 mM) was prepared in Milli-Q water, filtered through a 0.22 μm membrane filter, and diluted to a final working concentration of 20 μM prior to use. For each measurement, Hb samples were mixed with ThT to obtain a final dye concentration of 20 μM and incubated at room temperature for 45 mins for dye-protein interactions. The protocol was adapted from our previous study (Basha et al., 2026). Samples (200 μl per well) were transferred to black, flat-bottom 96-well microplates suitable for fluorescence measurements. Fluorescence spectra were recorded using a Tecan Spark multimode microplate reader (Männedorf, Switzerland) with excitation at 400 nm and emission collected between 450 and 800 nm. All measurements were performed in triplicate (N = 3), and mean values were used for analysis. To evaluate the influence of prolonged incubation on aggregate formation, a time-dependent ThT fluorescence study was performed using native Hb and Hb treated with 100 μM peroxynitrite. Following oxidant treatment, samples were incubated at 37 °C in sealed low-protein-binding, amber-tinted polypropylene microcentrifuge tubes. Aliquots were collected at 0, 12, 24, and 36 h and mixed with ThT as described above. Fluorescence intensity was monitored at 485 nm, corresponding to the characteristic emission maximum of ThT bound to β-sheet-rich aggregates. Changes in fluorescence intensity over time were used to assess the progression of aggregation during extended incubation under physiological temperature conditions. Measurements were performed in triplicate, and the mean fluorescence intensity at each time point was used for kinetic analysis.

1.6. **Congo Red**

Congo Red (CR) binding was used to evaluate the formation of aggregates consistent with increased structural ordering in native and peroxynitrite-treated hemoglobin (Hb) samples. A CR stock solution (500 μM) was prepared in Milli-Q water, filtered through a 0.22 μm membrane filter, and diluted to obtain a final working concentration of 20 μM prior to use. For each measurement, Hb samples were mixed with CR to achieve a final dye concentration of 20 μM and incubated at room temperature for 45 mins for dye-protein interactions. The protocol was adapted from our previous study (Basha et al., 2026). Samples (200 μl per well) were transferred to UV-transparent, black flat-bottom 96-well microplates. Absorption spectra were recorded using a Tecan Spark multimode microplate reader (Männedorf, Switzerland) over the wavelength range of 300–700 nm. All measurements were performed in triplicate (N = 3), and mean values were used for analysis. To evaluate the influence of prolonged incubation on aggregate formation, a time-dependent Congo Red binding study was performed using native Hb and Hb treated with 100 μM peroxynitrite. Following oxidant treatment, samples were incubated at 37 °C in sealed low-protein-binding, amber-tinted polypropylene microcentrifuge tubes. Aliquots were collected at 0, 12, 24, and 36 h and mixed with CR as described above. Absorbance was monitored at 500 nm, corresponding to the characteristic absorption region of Congo Red associated with protein aggregate binding. Changes in absorbance over time were used to assess the progression of aggregation during extended incubation under physiological temperature conditions. Measurements were performed in triplicate, and the mean absorbance value at each time point was used for kinetic analysis.

1.7. **8-anilinonaphthalene-1-sulfonic acid**

The 8-anilinonaphthalene-1-sulfonic acid (ANS) fluorescence assay was performed to evaluate changes in surface hydrophobicity and conformational transitions associated with peroxynitrite-modified hemoglobin (Hb). ANS was prepared as a 500 μM stock solution in Milli-Q water, filtered through a 0.22 μm membrane filter, and diluted to obtain a final working concentration of 50 μM prior to use. Hb samples were mixed with ANS and incubated at room temperature for 45 min to allow probe–protein interactions. The protocol was adapted from our previous study (Basha et al., 2026). Samples (200 μl per well) were transferred to black, flat-bottom 96-well microplates suitable for fluorescence measurements. Fluorescence spectra were recorded using a Tecan Spark multimode microplate reader (Männedorf, Switzerland), with excitation at 350 nm and emission collected between 400 and 800 nm. All measurements were performed in triplicate (N = 3), and mean values were used for analysis. To evaluate the influence of prolonged incubation on structural remodeling and hydrophobic surface exposure, a time-dependent ANS fluorescence study was performed using native Hb and Hb treated with 100 μM peroxynitrite. Following oxidant treatment, samples were incubated at 37 °C in sealed low-protein-binding, amber-tinted polypropylene microcentrifuge tubes. Aliquots were collected at 0, 12, 24, and 36 h, mixed with ANS as described above, and incubated for 45 min at room temperature prior to measurement. Fluorescence intensity was monitored at 520 nm, corresponding to the characteristic emission maximum of ANS bound to hydrophobic regions of proteins. Changes in fluorescence intensity over time were used to assess the progression of conformational changes and hydrophobic surface exposure during extended incubation. Measurements were performed in triplicate, and the mean fluorescence intensity at each time point was used for kinetic analysis.

1.8. **Fourier Transform Infrared Spectroscopic (FTIR)**

FTIR spectroscopy was employed to assess native and peroxynitrite-modified Hb. The lyophilized samples were recorded using a single-reflection diamond attenuated total reflectance (ATR) accessory on an FT/IR-4X spectrometer (JASCO Ltd., Tokyo, Japan) equipped with a mercury cadmium telluride (MCT) detector, which was used to record the spectra from lyophilized protein samples over a spectral range of 4000–600 cm⁻¹ to capture characteristic vibrational modes associated with protein secondary structures. Background spectra were subtracted to enhance the signal-to-noise ratio. Further, the transmittance FTIR spectra of native and peroxynitrite-modified Hb were converted to absorbance using equation 1, where A is absorbance, and T is transmittance (%). Further, a region of interest (1580 – 1720 cm^-1^) was selected and subsequently smoothened (to reduce the noise) using the Savitsky Golay algorithm, followed by unit-based normalization and baseline correction (Shah et al., 2026) using Spectragryph software (version 1.2.16.1). Secondary structure composition was quantified by deconvolution of the Amide I (1580–1720 cm⁻¹) using curve-fitting (Gaussian) to quantify: α-helix (~1650 –1660 cm⁻¹), β-sheet (~1610–1640 cm⁻¹ and 1680–1695 cm⁻¹), random coil (~1640–1650 cm⁻¹) and β-turns (~1660–1680 cm⁻¹) band using Gaussian curve fitting in OriginPro 2026, following previously reported studies (Basha et al., 2026; Mukhopadhyay et al., 2022).

$\boxed{A=-\log_{10}\left( \frac{T}{100} \right)}$………… **Eq. (1)**

The percentage contribution of each secondary-structural component was calculated using the following equation 2**.**

${\%}_{i}=\frac{A_{i}}{\Sigma A}\times100$………… **Eq. (2)**

Where Aᵢ represents the integrated area of the i^th^ Gaussian peak obtained from Amide I band deconvolution, and ΣA is the sum of areas of all fitted amide I components. This approach allows direct quantitative comparison of secondary-structure content between native and peroxynitrite-modified proteins and is widely accepted as the standard method for FTIR-based protein structural analysis (Mukhopadhyay et al., 2022).

1.9. **Scanning Electron Microscopy**

The surface morphology of lyophilized native and peroxynitrite-modified Hb samples was examined using a JEOL-IT300LV scanning electron microscope (JEOL, Ltd., Tokyo, Japan) operated at room temperature (25 °C). Each sample was mounted onto aluminum stubs with carbon adhesive tape and coated with a thin layer of gold via sputter coating to enhance conductivity and imaging quality. SEM images were acquired at multiple magnifications, enabling visualization and comparison of surface structural features between native and peroxynitrite-modified Hb (Basha et al., 2026).

1.10. **Dynamic Light Scattering Analysis**

The hydrodynamic size, polydispersity index (PDI), and zeta potential of native and peroxynitrite (PN)–modified hemoglobin (Hb) were determined using a Dynamic light scattering instrument (Nano‑ZS 90, Malvern Instruments). The refractive index of the protein was set to 1.45, the refractive index of the solvent (water) to 1.33, and an extinction coefficient of 0.960 was used for the calculations. For each Hb sample, three independent measurements were performed; in each measurement, 12 runs were acquired, and the instrument‑reported Z‑average hydrodynamic diameter and corresponding PDI were recorded. Zeta potential was also measured under identical buffer conditions, and the mean value from three independent measurements was used for data analysis. The experimental protocol was adapted from (Basha et al., 2026).

1.11. **Fluorescence Microscopy**

Fluorescence microscopy was used to visualize and compare aggregate formation in native and peroxynitrite-modified hemoglobin (Hb) samples. Protein samples were incubated with 20 μM thioflavin T (ThT) at 37 °C for 24 h to label β-sheet-rich and amyloid-like aggregate structures. Following incubation, 100 μl of each sample was placed onto a clean glass microscope slide, covered with a coverslip, and immediately examined using an inverted fluorescence microscope (CKX53, Olympus, Japan) equipped with an appropriate fluorescence filter set for ThT detection. Images were acquired using a 10X magnification with a 100 μM scale bar under identical acquisition settings to enable direct comparison between samples. Fluorescence microscopy was employed to assess aggregate morphology, distribution, and relative abundance following peroxynitrite treatment. Additional imaging procedures followed those described previously (Basha et al., 2026).

1.12. **Atomic Force Microscopy**

Atomic Force Microscopy (AFM) was carried out using a NanoWizard® 3 AFM system (JPK Instruments, Germany) to visualize morphological changes in native and peroxynitrite-modified hemoglobin (Hb). Protein samples were diluted 1:10 immediately before imaging. A 5 μl aliquot of each diluted sample was drop-cast onto freshly cleaved muscovite mica substrates (AFM-71856-03) and allowed to adsorb for 2 min, followed by gentle drying under ambient conditions. AFM imaging was performed in tapping mode using silicon cantilevers under ambient air. Topographical images were acquired over a scan area of 10 μm × 10 μm using identical imaging parameters for all samples. Height maps, three-dimensional rendered topography profiles, particle height distributions, and root mean square (RMS) roughness values were extracted and analyzed using JPK Data Processing software. Additional imaging procedures followed those described previously (Basha et al., 2026).

1.13. **X-ray powder diffraction (XRPD) Analysis**

X-ray powder diffraction (XRPD) patterns of native and peroxynitrite-modified hemoglobin (Hb) were recorded using a Malvern PANalytical® Empyrean Series 3 X-ray diffractometer. Diffraction scans were collected over a 2θ range of 5°–100° with a step size of 0.02° and a counting time of 1 s per step. Raw diffraction data were processed using Spectragryph v1.2.16.1, including baseline correction and normalization where appropriate, and the resulting diffractograms were plotted and visualized using OriginPro 2026 software (Basha et al., 2026).

1.14. **Spectral Data Processing**

The fluorescence spectrum of native and peroxynitrite-modified Hb was analyzed and pre-processed using SpectraGryph v1.2.16.1 software to assist in identifying the fluorescence peaks in the study. Subsequently, the spectra were collected without applying baseline correction. The region of interest (ROI) for each spectrum was chosen, spanning from 300 to 600 nm (Basha et al., 2026). The fluorescence spectra of extrinsic fluorescent dyes analysis of native and peroxynitrite-modified Hb recorded in the Tecan Spark multimode microplate reader (Mannedorf, Switzerland), XRPD, and FTIR are also taken for subsequent pre-processing using Spectragryph v1.2.16.1 software. Afterwards, the software Prism GraphPad version 8.0 and OriginPro 2026 are used to plot the graph of the pre-processed spectra.

1.15. **Statistical Analysis**

All quantitative data are presented as mean ± standard deviation (SD) from at least three independent measurements (N = 3). Statistical analyses were performed using GraphPad Prism version 8.0 (GraphPad Software, USA). Comparisons among experimental groups were evaluated using Brown–Forsythe and Welch one-way ANOVA where appropriate. Statistical significance was assessed at a confidence level of 95%. Significance levels are indicated as *P < 0.05 and ****P < 0.0001. Mean values, standard deviations, and preliminary calculations were obtained using Microsoft Excel 2019. Graphs and data visualizations were generated using GraphPad Prism version 8.0 and OriginPro 2026 (Origin Lab Corporation, USA).

2. **Results:**
   2.1. **Quantification of Peroxynitrite Stock Solution**

The concentration of the commercial sodium peroxynitrite stock solution (Cayman Chemical, Item No. 81565) was determined by UV–visible spectroscopy at 302 nm according to the manufacturer’s recommended procedure. The product is supplied as a solution in 0.3 M sodium hydroxide, and the concentration was verified immediately before use because peroxynitrite is unstable and gradually decomposes even during frozen storage.

a) **Sample preparation**

Peroxynitrite stock solution was thawed on ice and maintained under cold alkaline conditions throughout handling. A 40-fold dilution was prepared by mixing 25 μl of the stock solution with 975 μl of ice-cold 0.3 M NaOH, and absorbance was measured at 302 nm using 0.3 M NaOH as the blank. The extinction coefficient used for the calculation was ε302 = 1670 M^-1 cm^-1, as specified by the manufacturer.

b) **Raw absorbance values**

The absorbance readings obtained at 302 nm were as follows:

- Peroxynitrite diluted 40-fold (triplicates, wells B1–B3): 0.3709, 0.3697, 0.3422.
- Blank, 0.3 M NaOH (triplicates, wells B4–B6): 0.1090, 0.0767, 0.0666.

c) **Calculations**

The mean absorbance of the 40-fold diluted peroxynitrite sample was:

$$A_{\text{PN,raw}}=\frac{0.3709+0.3697+0.3422}{3}=0.3609$$

The mean absorbance of the blank was:

$$A_{\text{blank}}=\frac{0.1090+0.0767+0.0666}{3}=0.0841$$

The blank-corrected absorbance at 302 nm was therefore:

$$A_{302}=0.3609-0.0841=0.2768$$

Using Beer–Lambert’s law with a 1 cm pathlength cuvette:

$$c_{\text{diluted}}=\frac{A_{302}}{\varepsilon l}=\frac{0.2768}{1670\times1}=1.66\times{10}^{-4} \text{M}$$

Thus, the concentration of the 40-fold diluted sample was 0.166 mM. Because this sample was prepared by a 40-fold dilution of the original stock, the stock concentration was:

$$c_{\text{stock}}=40\times0.166 \text{mM}=6.64 \text{mM}$$

d) **Final result**

Based on the absorbance measurements and blank correction, the experimentally determined concentration of the sodium peroxynitrite stock used in this study was **6.64 mM**.

2.2. **Carbonyl Content**

**Table S1.** Carbonyl content in native and PN-treated Hb by DNPH assay.

| **Sample** | **A370 (+DNPH)** | **A370 (No DNPH)** | **Corrected A370** | **Carbonyl Content (nmol/mg)** |
| --- | --- | --- | --- | --- |
| Native Hb | -0.018 | 0.010 | −0.028 | 0.00 ± 0.00 |
| Hb + 20µM PN | -0.018 | -0.060 | +0.042 | 0.66 ± 0.02 |
| Hb + 100µM PN | 0.038 | -0.014 | +0.052 | 1.79 0.03 |

2.3. **Absorbance scan**

**Figure S1**. The absorbance of native Hb before conducting experiments. (a) absorbance scan native Hb at 200-500nm; (b) Absorbance of native Hb at 541nm; (c) Absorbance of native Hb at 577nm; and (d) Absorbance of native Hb at 630nm.

2.4. **Dynamic light scattering**

**Table S2.** Dynamic light scattering (DLS) measurements of native hemoglobin (Hb) and PN-treated Hb samples. The Z-average hydrodynamic diameter, polydispersity index (PDI) and Zeta potential (mV) are shown for three independent measurements of each sample.

| **Sample** | **Z‑average (d.nm)** | **PDI** | **Zeta potential (mV)** |
| --- | --- | --- | --- |
| Native Hb | 147.6 | 0.349 | −11.1 |
| Native Hb | 149.4 | 0.283 | −11.7 |
| Native Hb | 149.9 | 0.282 | −12.6 |
| Hb + 20 µM PN | 170.9 | 0.384 | −6.04 |
| Hb + 20 µM PN | 174.4 | 0.337 | −5.28 |
| Hb + 20 µM PN | 171.7 | 0.293 | −6.47 |
| Hb + 100 µM PN | 183.1 | 0.400 | −2.37 |
| Hb + 100 µM PN | 178.2 | 0.474 | −1.70 |
| Hb + 100 µM PN | 235.3 | 0.476 | −1.91 |

**Figure S2.** The Z-average hydrodynamic diameter and polydispersity index (PDI) of Native Hb, Hb with 20μM PN and Hb with 100μM PN were determined by Dynamic Light Scattering (DLS) based on three independent measurements.

**Figure S3.** The Zeta potential (mV) of Native Hb, Hb with 20μM PN and Hb with 100μM PN was determined by Dynamic Light Scattering (DLS) based on three independent measurements.

2.5. **Atomic Force Microscopy**

**Table S3.** Roughness measurements of Native Hb and Hb treated with Peroxynitrite.

| Samples | Average Roughness Ra | RMS Roughness Rq |
| --- | --- | --- |
| Native Hb | 61.97 nm | 80.36 nm |
| Hb + 20µM PN | 98.52 nm | 132.5 nm |
| Hb + 100µM PN | 238.2 | 325.8 |

**Figure S4.** The roughness measurements of Native Hb.

**Figure S5.** The roughness measurements of 20μM PN-treated Hb.

**Figure S6.** The roughness measurements of 100μM PN-treated Hb.

3. **References**

Arif, Z., Arfat, M. Y., Neelofar, K., Ahmad, S., Badar, A., Khan, M. A., Zaman, A., & Ahmad, J. (2017). Effect of peroxynitrite on human serum albumin: a multi technique approach. *Journal of Biomolecular Structure and Dynamics*, *35*(9), 2066–2076. https://doi.org/10.1080/07391102.2016.1206489

Basha, S., Mukunda, D. C., Chandra, S., Gangadharan, G., Patil, A., Pai, A. R., & Mahato, K. K. (2026). Label-free evaluation of protein aggregation by a point-of-care fluorometric device. *International Journal of Biological Macromolecules*, 150333. https://doi.org/10.1016/j.ijbiomac.2026.150333

Mukhopadhyay, A., Stoev, I. D., King, David. A., Sharma, K. P., & Eiser, E. (2022). Amyloid-Like Aggregation in Native Protein and its Suppression in the Bio-Conjugated Counterpart. *Frontiers in Physics*, *10*. https://doi.org/10.3389/fphy.2022.924864

Prabhu, V., Acharya, A., Satish Rao, B. S., Rathnakar, B., Kumar, P., Guddattu, V., & Mahato, K. K. (2018). Probing endogenous collagen by laser‐induced autofluorescence in burn wound biopsies: A pilot study. *Journal of Biophotonics*, *11*(9). https://doi.org/10.1002/jbio.201700394

Shah, P. U., Angadi, P. V., Mukunda, D. C., Karlo, J., & Singh, S. P. (2026). Raman spectroscopic detection of salivary thiocyanate for smoking exposure stratification. *The Analyst*. https://doi.org/10.1039/D6AN00372A
